# Roles of Hormones in Elevated pH-Mediated Mitigation of Copper Toxicity in *Citrus sinensis* Revealed by Targeted Metabolome

**DOI:** 10.3390/plants12112144

**Published:** 2023-05-29

**Authors:** Jiang Zhang, Wei-Lin Huang, Wei-Tao Huang, Xu-Feng Chen, Huan-Huan Chen, Xin Ye, Lin-Tong Yang, Li-Song Chen

**Affiliations:** Department of Resources and Environment, College of Resources and Environment, Fujian Agriculture and Forestry University, Fuzhou 350002, China; 2190807006@fafu.edu.cn (J.Z.); 2210807003@fafu.edu.cn (W.-L.H.); 1190807012@fafu.edu.cn (W.-T.H.); 2210807010@fafu.edu.cn (X.-F.C.); 2200807011@fafu.edu.cn (H.-H.C.); yexin1000@fafu.edu.cn (X.Y.); talstoy@fafu.edu.cn (L.-T.Y.)

**Keywords:** *Citrus sinensis* leaves and roots, copper–pH interaction, hormone, targeted metabolome

## Abstract

The effects of copper (Cu)–pH interactions on the levels of hormones and related metabolites (HRMs) in *Citrus sinensis* leaves and roots were investigated. Our findings indicated that increased pH mitigated Cu toxicity-induced alterations of HRMs, and Cu toxicity increased low-pH-induced alterations of HRMs. Increased pH-mediated decreases in ABA, jasmonates, gibberellins, and cytokinins, increases in (±)strigol and 1-aminocyclopropanecarboxylic acid, and efficient maintenance of salicylates and auxins homeostasis in 300 μM Cu-treated roots (RCu300); as well as efficient maintenance of hormone homeostasis in 300 μM Cu-treated leaves (LCu300) might contribute to improved leaf and root growth. The upregulation of auxins (IAA), cytokinins, gibberellins, ABA, and salicylates in pH 3.0 + 300 μM Cu-treated leaves (P3CL) vs. pH 3.0 + 0.5 μM Cu-treated leaves (P3L) and pH 3.0 + 300 μM Cu-treated roots (P3CR) vs. pH 3.0 + 0.5 μM Cu-treated roots (P3R) might be an adaptive response to Cu toxicity, so as to cope with the increased need for reactive oxygen species and Cu detoxification in LCu300 and RCu300. Increased accumulation of stress-related hormones (jasmonates and ABA) in P3CL vs. P3L and P3CR vs. P3R might reduce photosynthesis and accumulation of dry matter, and trigger leaf and root senescence, thereby inhibiting their growth.

## 1. Introduction

Like other heavy metals, micronutrient copper (Cu) has high phytotoxicity at high concentrations [1]. Currently, excessive Cu in soil is becoming a major factor limiting productivity in some old *Citrus* orchards due to long-term and heavy application of Cu-containing fungicides against fruit and foliar diseases and pests [2,3]. Since most Cu is preferentially immobilized in Cu-exposed roots, inhibition of root growth in response to Cu toxicity is usually prior to inhibition of shoot growth [4]. Root growth inhibition and functional damage caused by Cu toxicity in turn impairs water and nutrient uptake by roots, thereby reducing shoot growth [5,6,7].

Phytohormones, namely cytokinins (CKs), gibberellins (GAs), auxins (AUXs), abscisic acid (ABA), ethylene (ETH), jasmonates (JAs), salicylates (SAs), and strigolactones (SLs) play a role in plant Cu tolerance [8]. Evidence shows that exogenous supplement of ABA, gibberellin A_3_ (GA_3_), 1-naphthylacetic acid (1-NAA, a synthetic functionally analogue of auxin), indole-3-acetic acid (IAA), jasmonic acid (JA), salicylic acid (SA), 6-benzyladenine (BAP), and kinetin can mitigate Cu toxic inhibition on plant growth by reducing Cu uptake, inhibiting Cu translocation from roots to shoots, improving nutrient status, maintaining cellular redox homeostasis and/or preventing oxidative damage [9,10,11,12,13,14,15,16,17].

Cu toxicity had great impacts on hormone biosynthesis and concentrations in various plant tissues (organs). Matayoshi et al. [18] observed that Cu toxicity led to drastic decreases in the concentrations of JA, JA-isoleucine conjugate, IAA, ABA, GA_3_, and GA_4_ in maize (*Zea mays*) root apex, concluding that Cu toxicity-induced alterations of hormonal homeostasis at the root apex contributed to the strong root growth inhibition. Reckova et al. [19] reported that Cu-excessive maize roots had increased concentrations of SA, JA, ABA, and IAA, but unaltered concentration of active CKs; while Cu-excessive leaves had elevated concentrations of CKs, JA, IAA, and ABA, but unchanged concentration of SA. In *Citrus grandis* leaves, Cu excess led to increased levels of indole-3-lactic acid (ILA), IAA, ABA, L-tryptophan (TRP), cis-zeatin-9-glucoside (cZ9G), and total AUXs; and decreased levels of 5-deoxystrigol (5DS) and N6-benzyladenine-7-glucoside (BAP7G) [8]. Other reports indicated that Cu toxicity altered the concentrations of free and bound indole butyric acid (IBA) and IAA in radish (*Raphanus sativus*) hypocotyls [20]; trans-zeatin riboside (t-ZR), IAA and GA_3_ in *Arabidopsis thaliana* roots, and dihydrozeatin riboside (DHZR) and t-ZR in *A. thaliana* shoots [21]; ABA in PE19/66 (*Populus deltoides*), sunflower (*Helianthus annuus*), and maize leaves and roots [22,23,24]; and IAA in roots and ABA in leaves of B229 (*P. deltoids*) [23].

Evidence shows that high pH can counteract the adverse impacts of Cu toxicity on plants [2,25]. Most studies, however, have focused on investigating the impacts of Cu–pH interactions on plant growth; Cu and other nutrient uptake; root exudates; cell wall components, non-structural carbohydrates, gas exchange, gene expression and metabolite levels in leaves; and reactive oxygen species (ROS) and methylglyoxal (MG) formation and scavenging in leaves and roots [2,7,25,26,27]. Wu et al. [28] used targeted metabolome to investigate the impacts of aluminum (Al)–pH interactions on hormone levels in *Citrus sinensis* roots, concluding that increased pH-induced decrease in total CKs, and increases in IAA, SA, total JAs, JA and methyl jasmonate (MEJA) in Al toxic roots might confer Al-tolerance by reducing Al uptake, increasing the Al-triggered release of malate and citrate and Al sequestration in vacuole, and maintaining the homeostasis of nutrients and the balance between ROS and MG biosynthesis and removal. Thus, hormones might play a role in elevated pH-mediated mitigation of Cu toxicity in plants. Currently, such data are very rare.

*Citrus* are mainly planted in acid soils with high Cu bioavailability [29]. Here, we used targeted metabolome to explore Cu–pH interactions-induced alterations in the levels of hormones and related metabolites (HRMs) in *C. sinensis* leaves and roots. The objectives were (a) to elucidate how Cu–pH interactions affect the abundances of HRMs in leaves and roots, and (b) to test the hypothesis that hormones play a role in elevated pH-mediated amelioration of Cu toxicity.

## 2. Results

### 2.1. Profiles of HRMs in Leaves

We tested 88 HRMs in leaves (Table S1), 55 of which were detected in pH 3.0 + 0.5 μM Cu-treated leaves (P3L), pH 3.0 + 300 μM Cu-treated leaves (P3CL), pH 4.8 + 0.5 μM Cu-treated leaves (P5L), and/or pH 4.8 + 300 μM Cu-treated leaves (P5CL) (Table 1 and Figure S1).

We detected higher concentrations of iP7G, tZOG, 2MeScZR, DHZ7G, cZ9G, BAP7G, and total CKs, and lower concentrations of DHZR and IPR in P3CL vs. P3L; higher concentration of IPR in P5CL vs. P5L and P3L vs. P5L; and higher concentrations of iP7G, tZOG, DHZ7G, cZ9G, and total CKs, and lower concentrations of DHZR and IPR in P3CL vs. P5CL. BAP9G was detected only in P3CL and P5CL, and its level was similar between the two. K9G, IP, and cZ were not detected in P5L, P5L, and P3CL, respectively, and their levels were similar among the other three detected treatments (Table 1).

We observed higher concentrations of IAA-Glu, TRP, IAA, IAA-Trp, IAA-Val, MEIAA, ICAld, ILA, IAN, and total AUXs in P3CL vs. P3L; higher concentration of IAA-Trp in P5CL vs. P5L; lower concentration of IAA-Val in P3L vs. P5L; and higher concentrations of IAA-Glu, TRP, IAA-Trp, IAA, IAA-Val, MEIAA, ICAld, ILA, and total AUXs in P3CL vs. P5CL. IAA-Leu and IAGlc were not detected in P3CL and P5CL, respectively, and their concentrations did not significantly differ among the other three detected treatments. TRA and IAM were detected only in P3CL (Table 1).

We found higher concentration of JA-ILE, GA_24_, ABA, ABA-GE, total ABAs, SAG, SA, total SAs, ST, and total SLs, and lower concentrations of H2JA, OPDA, and 5DS in P3CL vs. P3L; higher concentration of JA-ILE in P5CL vs. P5L; higher concentrations of H2JA and OPDA in P3L vs. P5L; and higher concentrations of ABA, SAG, total SAs, ST, and total SLs, and lower concentrations of H2JA, JA-Val and 5DS in P3CL vs. P5CL (Table 1).

### 2.2. Profiles of HRMs in Roots

We tested 88 HRMs in roots (Table S2), 56 of which were detected in pH 3.0 + 0.5 μM Cu-treated roots (P3R), pH 3.0 + 300 μM Cu-treated roots (P3CR), pH 4.8 + 0.5 μM Cu-treated roots (P5R), and/or pH 4.8 + 300 μM Cu-treated roots (P5CR) (Table 2 and Figure S1).

We detected higher concentrations of 2MeSiPR, iP9G, cZROG, tZOG, IP, and total CKs, and lower concentrations of BAPR, IPR, and tZR in P3CR vs. P3R; higher concentration of iP9G, and lower concentrations of cZR, 2MeSiPR, BAPR, IPR, tZR, tZOG, and total CKs in P5CR vs. P5R; lower concentrations of BAPR, IPR, tZR, tZOG, and total CKs in P3R vs. P5R; and higher concentrations of 2MeSiPR, cZROG, tZOG, IP, and total CKs, and lower concentration of tZR in P3CR vs. P5CR. tZ and 2MeSiP were not detected in P5R, and their levels were higher in P3CR than in P3R and P5CR. K9G was not detected in P5CR, and its level was similar among P3R, P3CR, and P5R. BAP was not detected in P3R, and its level was higher in P5R than in P3CR and P5CR. BAP9G and cZ were not detected in P3R and P5R, and their levels were higher in P3CR than in P5CR. pT was not detected in P5R and P5CR, and its level did not significantly differ between P3R and P3CR. BAP7G was detected only in P5R (Table 2).

We found higher concentrations of OxIAA, TRA, ICA, IAA-Glu, TRP, IAA, IAA-Asp, MEIAA, ICAld, and total AUXs, and lower concentration of IAA-Val in P3CR vs. P3R; higher concentrations of OxIAA, IAA-Glu, IAA, and IAA-Asp, and lower concentrations of ICA and ICAld in P5CR vs. P5R; higher concentration of IAA-Val, and lower concentrations of ICA and IAN in P3R vs. P5R; and higher concentrations of TRA, ICA, TRP, IAA, MEIAA, ICAld, and total AUXs in P3CR vs. P5CR. IAA-Leu, IAA-Phe-Me, IAM, and IPA were detected only in P3R, P3CR, P5R, and P3CR, respectively (Table 2).

We observed higher concentrations of JA-ILE and OPC-4 in P3CR vs. P3R; lower concentrations of JA-ILE, OPDA, JA-Val, and total JAs in P5CR vs. P5R; lower concentrations of JA-ILE, JA, OPC-4, OPDA, JA-Val, and total JAs in P3R vs. P5R; and higher concentrations of JA-ILE in P3CR vs. P5CR. OPC-6 was not detected in P3CR and P5CR, and its level was similar between P3R and P5R (Table 2).

We detected higher concentrations of GA_24_ and GA_9_ in P3CR vs. P3R; lower concentrations of GA_1_ and total GAs in P5CR vs. P5R and P3R vs. P5R; and higher concentrations of GA_1_, GA_24_, GA_9_, and total GAs in P3CR vs. P5CR. GA_7_ was not detected in P5R and P5CR, and their levels did not significantly differ between P3R and P3CR (Table 2).

We observed higher concentrations of ABA, SAG, SA, and total SAs in P3CR vs. P3R; higher concentrations of ACC and ST, and lower concentration of ABA in P5CR vs. P5R; higher concentration of ACC, and lower concentrations of ABA and SA in P3R vs. P5R; and higher concentrations of ABA, SAG, and total SAs, and lower concentrations of ACC and ST in P3CR vs. P5CR (Table 2).

### 2.3. Principal Component Analysis (PCA) Uploading Plots

We used PCA to examine the responsive patterns of all HRMs detected in leaves and roots to Cu at pH 3.0 or 4.8 and to pH at 300 or 0.5 μM Cu. PC1 and PC2 contributed to 57.66% and 17.07%, 45.35% and 20.37%, 51.61% and 18.96%, and 41.31% and 21.71% of the total variation for pH 3.0-, pH 4.8-, 300 μM Cu-, and 0.5 μM Cu-treated leaves and roots, respectively (Tables S3–S6 and Figure S2A–S2D).

We also used PCA to examine the responsive patterns of all HRMs detected in leaves (55) and roots (56) to Cu–pH interactions. PC1 and PC2 accounted for 43.25% and 14.05% for leaves, and 39.35% and 21.62% for roots (Tables S7 and S8, Figure S2E,F).

## 3. Discussion

### 3.1. Cu toxicity and Low pH Displayed Synergistic Impacts on the Levels of HRMs in Leaves and Roots

We detected 25 upregulated HRMs, upregulated total CKs, total AUXs, total ABAs, total SAs, and total SLs, and seven downregulated HRMs in P3CL vs. P3L; 18 upregulated HRMs, upregulated total CKs, total AUXs, total SAs, and total SLs, and seven downregulated HRMs in P3CL vs. P5CL; six upregulated and one downregulated HRMs in P5CL vs. P5L; five upregulated and one downregulated HRMs in P3L vs. P5L; 28 upregulated HRMs, upregulated total CKs, total AUXs, and total SAs, and six downregulated HRMs in P3CR vs. P3R; 25 upregulated HRMs, upregulated total AUXs, total CKs, total GAs, and total SAs, and three downregulated HRMs in P3CR vs. P5CR; 11 upregulated HRMs, 17 downregulated HRMs, and downregulated total CKs, total JAs and total GAs in P5CR vs. P5R; and seven upregulated HRMs, 17 downregulated HRMs, and downregulated total CKs, total JAs and total GAs in P3R vs. P5R (Tables S9 and S10). These results indicated that low pH exacerbated Cu toxicity-induced alterations of HRMs levels in leaves and roots, and Cu toxicity exacerbated low pH-induced changes in HRMs levels in leaves and roots. Similarly, PCA indicated that Cu toxicity affected the levels of HRMs in leaves and roots more at pH 3.0 than those at pH 4.8, and low pH affected the levels of HRMs in leaves and roots more at 300 μM Cu than those at 0.5 μM Cu (Figure S2A–D). Collectively, Cu toxicity and low pH displayed synergistic impacts on the levels of HRMs in leaves and roots.

### 3.2. Hormones Involved in High pH-Mediated Alleviation of Cu Toxicity and Cu Excess-Mediated Exacerbation of Low pH Toxicity in Leaves and Roots

#### 3.2.1. AUXs in Leaves and Roots

We obtained upregulated total AUXs, TRA, IAM, TRP, IAA-Trp, IAA-Glu, MEIAA, IAA, ICAld, ILA, IAN, and IAA-Val, and downregulated IAA-Leu in P3CL vs. P3L; upregulated total AUXs, MEIAA, ICAld, IPA, TRA, IAA-Glu, IAA-Phe-Me, IAA, OxIAA, ICA, TRP, and IAA-Asp, and downregulated IAA-Val and IAA-Leu in P3CR vs. P3R; upregulated IAA-Trp and downregulated IAGlc in P5CL vs. P5L; and upregulated IAA-Glu, OxIAA, IAA-Asp, and IAA, and downregulated ICA, ICAld, and IAM in P5CR vs. P5R (Tables S9 and S10). Auxin homeostasis participates in the correct balance between cell proliferation and expansion since the highest IAA production site coincides with the cell division activity. Song et al. [13] found that Cu excess led to a dose-dependent reduction in IAA level in *A. thaliana* root tips. Exogenous application of 1-NAA mitigated Cu excess-induced reduction in primary root growth, while application of 1-N-naphthylphthalamic acid (NPA, an auxin efflux inhibitor) promoted Cu excess-induced reduction in primary root growth. Choudhary et al. [20] observed that Cu toxicity decreased (increased) free and bound IAA (IBA) concentrations in radish hypocotyls, concluding that Cu toxicity-induced decreases in free and bound IAA might be responsible for Cu toxicity-mediated inhibition of root and shoot growth. Pető et al. [30] observed that Cu toxicity elevated auxin levels in *A. thaliana* cotyledons and root apices, and decreased hypocotyl and primary root lengths and cotyledon area, concluding that endogenous hormonal balance and signal transduction played a role in Cu excess-triggered severe morphological responses. Sofo et al. [21] observed that Cu toxicity decreased *A. thaliana* root and shoot growth, increased IAA concentration in roots, and did not affect IAA concentration in shoots. Wang et al. [31] found that Cu toxicity inhibited root and shoot growth, and reduced auxin concentration in primary and lateral roots and leaf blade of *A. thaliana* seedlings. Wu et al. [8] identified one downregulated and four upregulated genes involved in auxin biosynthetic process; three downregulated and seven upregulated genes involved in auxin transport; and upregulated total AUXs, IAA, ILA, and TRP in Cu toxic *C. grandis* leaves, concluding that Cu toxicity-induced upregulation of auxin biosynthesis, transport, and levels might contribute to leaf Cu-tolerance by enhancing photosynthesis and water use efficiency (WUE). Ouzounidou and Ilias [17] found that supplement of 100 μM IAA alleviated Cu toxicity-induced decreases in root and shoot growth, chlorophylls, photosynthesis, and WUE in sunflower plants. Ben Massoud et al. [32] observed that application of IAA in the germination medium mitigated pea (*Pisum sativum*) seedlings Cu toxicity by decreasing Cu concentrations in shoots and roots and providing a thiol redox state to protect the proteins against oxidation. In *Brassica juncea*, foliar spray of IAA-mediated alleviation of Cu toxicity involved the increase in antioxidant capacity and the decreases in ROS, malondialdehyde (MDA), and electrolyte leakage levels in Cu toxic plants. Additionally, IAA application can improve plant nutrient status; maintain leaf function and photosynthesis; reduce root cell death; and ultimately increase Cu toxic plant biomass [9]. Yuan et al. [33] showed that Cu toxicity led to higher auxin levels in both the elongation and meristem zones, and a lower auxin level in columella cells of *A. thaliana* roots, which might contribute to Cu toxicity-induced inhibition of primary root elongation. TRP can act as a precursor for the biosynthesis of IAA and melatonin, which play a role in plant Cu-tolerance [34]. Collectively, Cu toxicity-induced increase in total AUXs (IAA) in pH 3.0-treated leaves and roots might be caused by enhanced biosynthesis due to increased substrate (TRP), and by less dilution due to reduced growth [2]. The increase in total AUXs (IAA) might be an adaptive response to Cu toxicity, but it did not protect pH 3.0-treated leaves and roots from Cu toxicity. Additionally, Cu toxicity might impair auxin homeostasis (balance) in pH 3.0-treated leaves and roots, thereby inhibiting their growth. Elevated pH lessened Cu toxicity-induced alterations of AUXs in leaves and roots, thus maintaining auxin homeostasis (balance) and mitigating Cu toxicity-induced inhibition of leaf and root growth.

We detected upregulated total AUXs, IAGlc, TRA, IAM, TRP, IAA-Glu, MEIAA, ILA, IAA, IAA-Trp, ICAld, and IAA-Val, and downregulated IAA-Leu in P3CL vs. P5CL; upregulated total AUXs, IAA, ICA, TRA, MEIAA, ICAld, IPA, IAA-Phe-Me, and TRP in P3CR vs. P5CR; downregulated IAA-Val in P3L vs. P5L; and upregulated IAA-Leu and IAA-Val, and downregulated IAN, ICA, and IAM in P3R vs. P5R (Tables S9 and S10), suggesting that low pH increased auxin biosynthesis and accumulation, and impaired auxin homeostasis in 300 μM Cu-treated leaves (LCu300) and 300 μM Cu-treated roots (RCu300), thereby lowering their growth, but less in 0.5 μM Cu-treated leaves (LCK) and 0.5 μM Cu-treated roots (RCK).

#### 3.2.2. CKs in Leaves and Roots

As shown in Tables S9 and S10, we detected upregulated total CKs, BAP9G, tZOG, DHZ7G, 2MeScZR, iP7G, cZ9G, and BAP7G, and downregulated IPR, DHZR, and cZ in P3CL vs. P3L; upregulated total CKs, BAP9G, tZOG, BAP, cZ, 2MeSiP, IP, iP9G, tZ, 2MeSiPR, and cZROG, and downregulated IPR, BAPR, and tZR in P3CR vs. P3R; upregulated BAP9G, K9G, IP, and IPR in P5CL vs. P5L; and upregulated BAP9G, cZ, 2MeSiP, tZ, and iP9G, and downregulated total CKs, tZOG, BAP, 2MeSiPR, tZR, cZR, IPR, BAPR, K9G, and BAP7G in P5CR vs. P5R. Werner et al. [35] observed that CK-deficient tobacco (*Nicotiana tabacum*) plants had decreased shoot (leaf) growth, but enhanced root growth, concluding that CKs played an opposite role in regulating shoot and root growth. Foliar application of two CKs (kinetin and BAP) conferred castor (*Ricinus communis*) Cu-tolerance by reducing Cu level in shoots and improving antioxidant ability [10]. Observations of β-glucuronidase reporter lines suggested that 50 μM Cu-induced increment of CK pool in *A. thaliana* roots might contribute to Cu toxicity-induced inhibition of root growth [36]. Sofo et al. [21] reported that Cu toxicity elevated the levels of tZR and DHZR in shoots and DHZR in roots of *A. thaliana* seedlings. Massot et al. [37] observed a rapid increment in CK level in Al-treated bean (*Phaseolus vulgaris*) prior to Al-induced inhibition of root growth. Al-induced rapid increment of CKs might contribute to root growth inhibition either directly or indirectly by influencing hormone homeostasis. Plant metallothioneins (MTs) have been shown to play a role in Cu-tolerance by lowering Cu toxicity-induced oxidative damage and binding Cu [38]. Thomas et al. [38] suggested that CKs can stimulate metallothionein-like gene mRNA transcription and/or stability. Under Cu-stress, Cu accumulation in older leaves with enhanced CK production preserved the younger growing vegetative tissues, which was conducive to plant survival. Transgenic plants with enhanced CK level displayed less lipid peroxidation. Our finding that Cu toxicity led to increased total CKs concentration in pH 3.0-treated leaves, but not in pH 4.8-treated leaves agreed with the report that Cu toxicity-induced increment of MT level in leaves was greater at pH 3.0 than that at pH 4.8, and its level was higher in P3CL than in P5CL [26]. The elevated accumulation of MTs in P3CL vs. P5CL agreed with the increased need for Cu and ROS detoxification, because Cu toxicity-induced accumulation of Cu and production of ROS in leaves were greater at pH 3.0 than those at pH 4.8 [2,26]. In *C. sinensis* seedlings, Al toxicity led to increased and decreased total CKs concentrations in pH 3.0- and pH 4.0-treated roots, respectively. Increased pH lowered the accumulation of CKs in roots, thereby ameliorating Al toxicity-induced inhibition of root growth [28]. Taken together, Cu toxicity increased total CKs level in pH 3.0-treated roots, thus lowering root growth, while Cu toxicity reduced total CKs level in pH 4.8-treated roots, thus mitigating Cu toxicity-induced root growth inhibition [2]. Cu toxicity increased total CKs level in pH 3.0-treated leaves, thus impairing hormone homeostasis and reducing leaf growth [2], while increased pH enhanced the capacity of LCu300 to maintain CKs homeostasis, thus improving leaf Cu-tolerance.

We detected upregulated total CKs, DHZ7G, iP7G, cZ9G, and tZOG, and downregulated DHZR, IPR, and cZ in P3CL vs. P5CL; upregulated total CKs, pT, K9G, 2MeSiP, BAP9G, cZ, IP, 2MeSiPR, tZOG, tZ, and cZROG, and downregulated tZR in P3CR vs. P5CR; upregulated K9G, IP, and IPR in P3L vs. P5L; and upregulated 2MeSiP, tZ and pT, and downregulated total CKs, tZR, BAPR, tZOG, IPR, BAP, and BAP7G in P3R vs. P5R (Tables S9 and S10). This agreed with the report that low pH increased and decreased total CKs in 1.0 and 0 mM Al-treated *C. sinensis* roots [28], and that zeatin riboside (ZR) concentration in *Atractylodes lancea* roots was greater at pH 5.3 than at pH 6.0 [39]. These findings suggested that low pH increased total CKs concentration in RCu300, thereby lowering root growth, but it decreased total CKs concentration in RCK, and did not significantly affect root growth [2]. Cu toxicity enlarged low pH impacts on CK homeostasis in leaves, thereby lowering leaf Cu-tolerance.

#### 3.2.3. JAs in Leaves and Roots

As shown in Tables S9 and S10, we detected upregulated JA-ILE, and downregulated H2JA and OPDA in P3CL vs. P3L; upregulated OPC-4 and JA-ILE, and downregulated OPC-6 in P3CR vs. P3R; upregulated JA-ILE in P5CL vs. P5L; and downregulated total JAs, JA-Val, OPDA, and OPC-6 in P5CR vs. P5R. Maksymiec and Krupa [40] observed that both Cu toxicity and MEJA inhibited *Phaseolus coccineus* and *Allium cepa* root growth, and maize leaf growth, and that JA synthesis inhibitors-namely ibuprofen (IB) and salicylhydroxamic acid (SHAM), mitigated Cu toxicity-induced growth inhibition in *P. coccineus* roots and maize leaves, but not in *A. cepa* roots. Using metabolome and transcriptome analyses, Hu et al. [41] demonstrated that melatonin alleviated Cu toxicity-induced growth inhibition of melon (*Cucumis melo*) via repressing JA biosynthesis. Taken together, increased pH prevented Cu toxicity-induced changes in JAs in leaves, thus alleviating leaf Cu toxicity; while Cu toxicity lowered the levels of JAs in pH 4.8-treated roots, thus improving root growth.

We detected downregulated H2JA and JA-Val in P3CL vs. P5CL; upregulated JA-ILE in P3CR vs. P5CR; upregulated H2JA and OPDA in P3L vs. P5L; and downregulated total JAs, JA-ILE, OPC-4, JA-Val, JA, and OPDA in P3R vs. P5R (Tables S9 and S10), suggesting that low pH reduced and elevated the concentrations of JAs in LCu300 and RCu300, respectively.

#### 3.2.4. ABAs in Leaves and Roots

As shown in Tables S9 and S10, we detected upregulated total ABAs, ABA, and ABA-GE in P3CL vs. P3L; upregulated ABA in P3CR vs. P3R; and downregulated ABA in P5CR vs. P5R. Cu toxicity-induced increment of ABA concentrations has been reported on radish seedlings [20], *C. grandis* leaves [8], and maize leaves and roots [22]. Cu excess-induced increase in ABA concentrations in sunflower roots, shoots, leaves, and seedlings was dose-dependent [24]. Kebert et al. [23] investigated Cu toxic impacts on ABA concentrations in the leaves and roots of three poplar clones, M1 (*Populus x euramericana*), B229 and PE19/66. Cu toxicity increased ABA concentrations in PE19/66 leaves and roots, and in B229 leaves, but had no significant impacts on ABA concentrations in M1 leaves and roots, and in B229 roots. Obviously, Cu toxicity impacts on ABA concentrations depended on Cu concentration, pH, tissue (organ), and genotype.

ABA can increase non-photochemical quenching (NPQ), the first line of defense to protect photosystem II (PSII) reaction centers against photo-oxidative damage [42]. The upregulation of ABA in P3CL vs. P3L implied that NPQ was upregulated in order to cope with the increased need for thermal dissipation, as indicated by increased quantum yield for dissipated energy (DI_o_/ABS) in P3CL vs. P3L, but not in P5CL vs. P5L [2]. The increase in ABA in P3CL vs. P3L might lead to stomatal closure which in turn reduced photosynthesis and accumulation of dry matter [22]. Bilal et al. [43] indicated that endophytic *Penicillium funiculosum* LHL06 mitigated the synergistic toxicity of heavy metals on soybean (*Glycine max*) by reducing uptake of heavy metals, accumulation of ABA and JA, and oxidative damage due to decreased H_2_O_2_ accumulation and enhanced antioxidant system in roots. Zehra et al. [12] reported that foliar spraying of ABA ameliorated Cu toxicity-induced decreases in plant growth, and leaf oxidative damage due to reduced ROS accumulation and elevated antioxidant enzyme activities. Additionally, ABA can downregulate the expression of light-harvesting chlorophyll a/b-binding proteins of PSII (Lhcb) gene, thereby protecting chloroplast from photooxidative damage and photoinhibition [42]. A study indicated that Cu toxicity-induced increase in superoxide anion production rates was higher in pH 3.0-treated than in pH 4.8-treated leaves and roots, and Cu toxicity increased H_2_O_2_ production rates in pH 3.0-treated leaves and roots, but not in pH 4.8-treated leaves and roots [26]. Taken together, the elevated concentration of ABA in P3CL vs. P3L agreed with the increased need for ROS detoxification.

We detected upregulated ABA in P3CL vs. P5CL; upregulated ABA in P3CR vs. P5CR; and downregulated ABA in P3R vs. P5R (Tables S9 and S10). The upregulation of ABA in P3CL vs. P5CL and P3CR vs. P5CR might contribute to low pH-induced stomatal closure, photosynthesis decline, and growth inhibition. Yan et al. [44] reported that maize was more tolerant to low pH than broad bean (*Vicia faba*). The critical pH, below which net H^+^ exudation and root growth ceased, were 3.5 for maize and 4.00 for broad bean at 1 mM Ca^2+^. With the decrease in pH of the medium, both H^+^ exudation and root growth decreased gradually. Additional ABA in root medium led to increased H^+^ exudation and root growth for maize at pH 4.0, but decreased H^+^ exudation and root growth for broad bean at pH 4.1. Yang et al. [45] observed that Al-stimulated exudation of malate and citrate in *C. sinensis* roots decreased with the decrease in pH. Taken together, low pH increased ABA concentration and decreased H^+^ exudation in RCu300, thus reducing root growth, but not in RCK. This agreed with the report that low pH affected root growth more at 300 μM Cu than at 0.5 μM Cu [2].

#### 3.2.5. SAs in Leaves and Roots

As shown in Tables S9 and S10, Cu toxicity elevated the levels of SAG, SA and total SAs in leaves and roots at pH 3.0, but not at pH 4.8. Evidence shows that application of SA can counteract the inhibitory action of Cu toxicity on growth of *Salvia officinalis* [46], bean [47], rice (*Oryza sativa*) [16], sunflower [48], and cotton (*Gossypium* spp.) [14] by reducing Cu translocation from roots to shoots, and Cu levels and oxidative damage in leaves and roots; and improving nutrient status and relative water content in leaves and roots, and chlorophyll and carotenoid levels in leaves. These results suggested that the increased accumulation of SA, SAG, and total SAs in P3CL vs. P3L and P3CR vs. P3R was an adaptive strategy to Cu toxicity. However, increased accumulation of SA might induce leaf and root senescence, thereby inhibiting their growth [49,50].

We detected upregulated SAG and total SAs in P3CL vs. P5CL and P3CR vs. P5CR, and downregulated SA in P3R vs. P5R (Tables S9 and S10), suggesting that low pH increased SAG and total SAs accumulation in RCu300 and LCu300, thereby inhibiting their growth.

#### 3.2.6. GAs in Leaves and Roots

As shown in Tables S9 and S10, we detected upregulated GA_24_ in P3CL vs. P3L, upregulated GA_9_ and GA_24_ in P3CR vs. P3R, and downregulated GA_1_ and total GAs in P5CR vs. P5R. Saleem et al. [51] observed that foliar spraying of GA_3_ mitigated *Corchorus capsularis* Cu toxicity by enhancing growth and photosynthesis, and reducing oxidative damage. Additionally, GA_3_ increased Cu accumulation in roots, stems, and leaves. Similar results have been obtained for pea [52] and sunflower [17]. Bioactive GA_4_ is biosynthesized from GA_24_ via GA_9_ [53]. Thus, the upregulation of GA_24_ in P3CL vs. P3L and GA_24_ and GA_9_ in P3CR vs. P3R might be an adaptive strategy to Cu toxicity, while the downregulation of GA_1_ and total GAs in P5CR vs. P5R and unaltered GAs in P5CL vs. P5L agreed with the finding that raised pH lessened Cu accumulation in Cu toxic roots, stems, and leaves [2].

As shown in Tables S9 and S10, we detected upregulated GA_7_, GA_1_, GA_24_, GA_9_, and total GAs in P3CR vs. P5CR, and upregulated GA_7_ and downregulated GA_1_ and total GAs in P3R vs. P5R. The upregulation of GA_7_, GA_1_, GA_24_, GA_9_, and total GAs in P3CR vs. P5CR might be an adaptive response to low pH, while the downregulation of GA_1_ and total GAs in P3R vs. P5R agreed with the report that low pH increased Cu concentrations in roots, stems and leaves less under 0.5 μM Cu than under 300 μM Cu [2].

#### 3.2.7. ETH in Roots

We obtained upregulated ACC in P5CR vs. P5R (Table S10). Flora et al. [54] showed that silicon-mediated amelioration of Cu toxicity in tobacco involved enhanced expression of ethylene biosynthetic genes. Masood et al. [55] found that cadmium (Cd)-induced evolution of ethylene in mustard (*Brassica juncea*) was reduced by ethephon (ethylene source) and sulfur, which resulted in reduced oxidative damage, upregulated antioxidant system, and elevated photosynthesis. Both ethephon- and sulfur-treated plants displayed more tolerance to Cd. S-mediated alleviation can be reversed by aminoethoxyvinylglycine (ethylene biosynthesis inhibitor). Thao et al. [56] suggested that the application of ethylene modulators for optimizing ethylene level was a wise strategy to confer heavy metal tolerance with minimal side effects. Taken together, increased pH might enhance the biosynthesis of ethylene, thus alleviating Cu toxicity.

We identified downregulated ACC in P3CR vs. P5CR, and upregulated ACC in P3R vs. P5R (Table S10), suggesting that Cu toxicity intensified low pH inhibition on root growth by reducing ethylene level in P3CR vs. P5CR.

#### 3.2.8. SLs in Leaves and Roots

We detected upregulated ST in P5CR vs. P5R and downregulated ST in P3CR vs. P5CR (Table S10). Qiu et al. [57] found that strigol analogue (GR24) conferred barley (*Hordeum vulgare*) Cd-tolerance by reducing Cd uptake and oxidative damage due to reduced H_2_O_2_ accumulation and elevated concentrations of ascorbate and reduced glutathione and activities of antioxidant enzymes in leaves and roots. Mostofa et al. [58] indicated that strigolactone-deficient rice mutant *d10* and *d17* displayed less tolerance to arsenic stress accompanied by increased arsenic concentration, decreased sequestration of arsenic in vacuole, and enhanced oxidative damage in roots. The upregulation of ST in P5CR vs. P5R and the downregulation of ST in P3CR vs. P5CR agreed with the findings that increased pH-mediated amelioration of Cu toxicity involved reduced Cu uptake and oxidative damage in roots, and that low pH caused oxidative damage and a greater increase in Cu uptake in RCu300, but not in RCK [2,26]. However, we detected upregulated ST and total SLs and downregulated 5DS in P3CL vs. P3L and P3CL vs. P5CL (Table S9), indicating that increased pH lessened Cu toxic impacts on SL levels, and that Cu toxicity intensified Cu toxic impact on SL levels.

## 4. Materials and Methods

### 4.1. Plant Materials

Plant material culture and treatments referred to Zhang et al. [26]. Six weeks after seed germination, uniform sweet orange (*Citrus sinensis* (L.) Osbeck cv. Xuegan) seedlings were transplanted to 6 L pots (two seedlings per pot) containing sand, and then cultivated in a greenhouse under the natural conditions at Fujian Agriculture and Forestry University, Fuzhou with an annual average relative humidity, temperature, and sunlight of ~ 76%, 20 °C and 1600 h, respectively [59]. After six weeks of seedling transplantation, seedlings were supplied with nutrient solutions six times weekly with 300 (Cu toxicity) or 0.5 (non-Cu toxicity or control) μM CuCl_2_ × pH 3.0 or 4.8 (adjusted by 1M HCl) until dripping (~500 mL per pot). Cu concentrations and pH levels of solutions were chosen based on our previous reports [2,26]. Each treatment had 40 seedlings (20 pots) in a completely randomized design. After 17 weeks of Cu–pH interaction treatments, about 5 mm in length of white root tips and the recent fully expanded (~seven weeks-old) leaves were collected at noon on a sunny day and immediately frozen in liquid N_2_, and then stored in a −80 °C freezer until extraction of HRMs.

### 4.2. Extraction and Assay of HRMs in Leaves and Roots

Equal amounts of frozen leaves (roots) from five seedlings from different pots were mixed as one biological replicate. There were three biological replicates per treatment. Samples were sent to Wuhan MetWare Biotechnology Co., Ltd. (https://www.metware.cn/, accessed on 1 June 2022) for assay of hormones. After frozen samples were ground into powder in liquid N_2_, 50 mg of the powder was transferred to 2 mL tube containing 1 mL of methanol:water:formic acid (15:4:1, v:v:v) and ten μL of internal standard mixed solution (100 ng mL^−1^). After 10 min of vortex, the mixture was centrifuged at 16,000× *g* for 5 min at 4 °C. The yielding supernatant was evaporated to dryness in N_2_ flow, dissolved in 100 μL of 80% methanol (*v*/*v*), and then filtered through a 0.22 μm filter. The filtrate was used for HRMs assay.

The concentrations of HRMs in the filtrate were determined using an ultra-performance liquid chromatography-electrospray ionization-tandem mass spectrometry (UPLC-ESI-MS/MS) system (UPLC, ExionLC™ AD, https://sciex.com.cn/, accessed on 1 June 2022; MS, Applied Biosystems 6500 Triple Quadrupole, https://sciex.com.cn/, accessed on 1 June 2022) [28].

### 4.3. Data Analysis

Data were analyzed by two-way ANOVA (two (Cu levels) × two (pH levels)), followed by the least significant difference (LSD) at *p* < 0.05 using DPS 7.05 (Hangzhou RuiFeng Information Technology Co., Ltd., Hangzhou, China). PCA was made with SPSS statistical software (version 17.0, IBM, New York, NY, USA).

## 5. Conclusions

**Figure 1:**
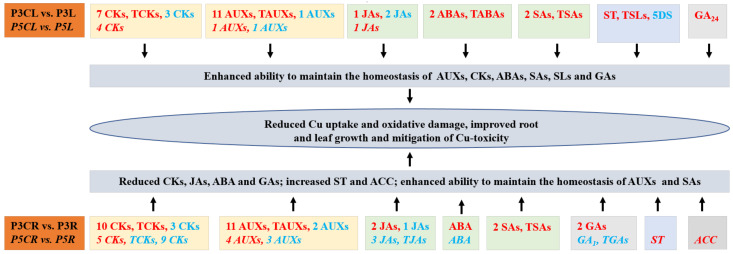
A model for the roles of hormones in elevated pH-mediated mitigation of Cu toxicity in leaves and roots. Red: upregulation; Blue: downregulation; TCKs: total CKs; TAUXs: total AUXs; TABAs: total ABAs; TJAs: total JAs; TSAs: total SAs; TGAs: total GAs; TSLs: total SLs.

Our results showed that elevated pH prevented the alterations of HRMs levels caused by Cu toxicity, while Cu toxicity aggravated the changes in HRMs levels caused by low pH. A model for the role of hormones in elevated pH-mediated mitigation of Cu toxicity in leaves and roots was proposed (Figure 1). Elevated pH-mediated decreases in CKs, ABA, JAs, and GAs, increases in ST and ACC, and efficient maintenance of SAs and AUXs homeostasis in RCu300; as well as efficient maintenance of hormone homeostasis in LCu300 might contribute to improved leaf and root growth, which can be partially explained by reduced Cu uptake and oxidative damage. The upregulation of AUXs (IAA), CKs, GAs, ABA, and SAs in P3CL vs. P3L and P3CR vs. P3R agreed with the increased need for ROS and Cu detoxification in LCu300 and RCu300, which might be an adaptive response to Cu toxicity. The elevated levels of stress-related hormones (JAs and ABA) in P3CL vs. P3L and P3CR vs. P3R might lead to less photosynthesis and accumulation of dry matter, and induce leaf and root senescence, thereby reducing their growth. Although this study demonstrated that raised pH-mediated decreases in CKs, JAs, GAs, and ABA, increases in ST and ACC, and homeostasis maintenance of AUXs and SAs in RCu300, as well as homeostasis maintenance of hormones in LCu300 played a key role in improved pH-induced alleviation of *Citrus* Cu toxicity, the mechanisms by which different hormones alleviate Cu toxicity are unclear and require further research.

## Figures and Tables

**Table 1:** Impacts of Cu–pH interactions on mean (±SE, *n* = 3) concentrations (ng g^−1^ FW) of hormones and related metabolites (HRMs) detected in *Citrus sinensis* leaves.

Hormones and Related Metabolites		Treatments		
	P3L	P3CL	P5L	P5CL
Cytokinins (CKs)				
Kinetin-9-glucoside (K9G)	0.137 ± 0.137 a	0.461 ± 0.461 a	ND	0.159 ± 0.159 a
N6-Benzyladenine-9-glucoside (BAP9G)	ND	0.053 ± 0.053 a	ND	0.034 ± 0.034 a
ortho-Topolin riboside (oTR)	1.189 ± 0.145 ab	0.559 ± 0.281 b	1.634 ± 0.255 a	1.200 ± 0.246 ab
Dihydrozeatin ribonucleoside (DHZR)	1.249 ± 0.238 a	0.661 ± 0.136 b	1.221 ± 0.124 a	1.227 ± 0.068 a
N6-Isopentenyl-adenine-7-glucoside (iP7G)	0.571 ± 0.035 b	2.392 ± 0.184 a	0.646 ± 0.034 b	0.653 ± 0.056 b
N6-isopentenyladenosine (IPR)	1.340 ± 0.312 a	0.399 ± 0.083 b	0.545 ± 0.063 b	1.225 ± 0.135 a
trans-Zeatin-O-glucoside (tZOG)	23.48 ± 1.47 b	31.36 ± 0.52 a	25.63 ± 1.66 b	24.40 ± 0.41 b
2-Methylthio-cis-zeatin riboside (2MeScZR)	0.025 ± 0.025 b	0.106 ± 0.008 a	0.022 ± 0.022 b	0.063 ± 0.010 ab
N6-isopentenyladenine (IP)	0.018 ± 0.009 a	0.033 ± 0.033 a	ND	0.011 ± 0.011 a
Dihydrozeatin-7-glucoside (DHZ7G)	0.179 ± 0.011 c	2.106 ± 0.306 a	0.328 ± 0.048 bc	0.461 ± 0.066 b
cis-Zeatin (cZ)	0.096 ± 0.051 a	ND	0.167 ± 0.027 a	0.053 ± 0.053 a
cis-Zeatin-9-glucoside (cZ9G)	1.394 ± 0.179 b	4.363 ± 0.490 a	1.291 ± 0.290 b	1.515 ± 0.140 b
N6-Benzyladenine-7-glucoside (BAP7G)	0.153 ± 0.031 b	0.298 ± 0.072 a	0.214 ± 0.007 ab	0.180 ± 0.018 ab
Total CKs	32.33 ± 1.05 b	45.24 ± 1.03 a	34.21 ± 1.38 b	34.02 ± 0.66 b
Auxins (AUXs)				
N-(3-Indolylacetyl)-L-leucine (IAA-Leu)	1.348 ± 0.065 a	ND	1.577 ± 0.087 a	1.522 ± 0.167 a
Tryptamine (TRA)	ND	0.321 ± 0.076	ND	ND
Indole-3-acetyl glutamic acid (IAA-Glu)	0.517 ± 0.265 b	1.822 ± 0.433 a	0.817 ± 0.423 ab	0.508 ± 0.265 b
1-O-indol-3-ylacetylglucose (IAGlc)	25.51 ± 12.78 a	41.09 ± 24.90 a	46.13 ± 23.15 a	ND
3-Indole acetamide (IAM)	ND	0.892 ± 0.892	ND	ND
L-tryptophan (TRP)	2089 ± 254 b	17494 ± 1124 a	2305 ± 193 b	1854 ± 73 b
Indole-3-acetyl-L-tryptophan (IAA-Trp)	0.598 ± 0.051 c	2.356 ± 0.328 a	0.549 ± 0.069 c	1.506 ± 0.114 b
Indole-3-acetic acid (IAA)	5.418 ± 0.098 b	11.853 ± 1.333 a	6.083 ± 0.792 b	7.052 ± 0.959 b
N-(3-Indolylacetyl)-L-valine (IAA-Val)	2.366 ± 0.011 c	3.531 ± 0.080 a	3.258 ± 0.132 ab	3.072 ± 0.085 b
Methyl indole-3-acetate (MEIAA)	0.262 ± 0.032 b	0.586 ± 0.049 a	0.325 ± 0.046 b	0.329 ± 0.025 b
Indole-3-carboxaldehyde (ICAld)	33.75 ± 0.53 b	65.51 ± 4.54 a	36.26 ± 0.87 b	42.06 ± 2.69 b
Indole-3-lactic acid (ILA)	16.13 ± 0.80 b	30.55 ± 1.00 a	19.31 ± 0.81 b	17.70 ± 1.33 b
3-Indoleacetonitrile (IAN)	1.693 ± 0.141 b	2.967 ± 0.595 a	1.457 ± 0.323 b	1.796 ± 0.268 ab
Total AUXs	2223 ± 262 b	17701 ± 1153 a	2474 ± 172 b	1975 ± 78 b
Jasmonates (JAs)				
Jasmonoyl-L-isoleucine (JA-ILE)	7.422 ± 0.764 b	25.321 ± 5.471 a	10.379 ± 4.166 b	30.813 ± 4.151 a
Dihydrojasmonic acid (H2JA)	2.343 ± 0.069 a	1.545 ± 0.142 c	1.708 ± 0.100 bc	2.168 ± 0.242 ab
cis(+)-12-Oxophytodienoic acid (OPDA)	166.33 ± 15.09 a	71.94 ± 10.67 b	99.61 ± 14.27 b	107.17 ± 17.25 b
N-[(−)-Jasmonoyl]-(L)-valine (JA-Val)	0.601 ± 0.018 ab	0.393 ± 0.105 b	0.753 ± 0.337 ab	1.143 ± 0.088 a
Gibberellins (GAs)				
Gibberellin A_24_ (GA_24_)	5.694 ± 0.188 b	7.597 ± 0.891 a	5.692 ± 0.243 b	6.091 ± 0.190 ab
Abscisates (ABAs)				
Abscisic acid (ABA)	13.61 ± 1.14 b	72.18 ± 2.95 a	10.95 ± 0.45 b	15.53 ± 0.59 b
ABA-glucosyl ester (ABA-GE)	185.2 ± 101.7 b	521.1 ± 57.5 a	315.9 ± 48.4 ab	475.3 ± 60.6 a
Total ABAs	198.9 ± 101.0 c	593.3 ± 59.1 a	326.9 ± 48.2 bc	490.8 ± 61.0 ab
Salicylates (SAs)				
Salicylic acid 2-O-β-glucoside (SAG)	23.15 ± 2.61 b	187.55 ± 40.93 a	33.30 ± 5.57 b	33.67 ± 2.13 b
Salicylic acid (SA)	75.23 ± 7.59 b	111.18 ± 12.72 a	82.49 ± 5.73 b	86.71 ± 4.43 ab
Total SAs	98.38 ± 10.18 b	298.73 ± 33.54 a	115.80 ± 6.11 b	120.38 ± 4.36 b
Strigolactones (SLs)				
(±)Strigol (ST)	107.2 ± 4.2 b	238.8 ± 18.0 a	126.6 ± 4.9 b	122.8 ± 18.6 b
5-Deoxystrigol (5DS)	12.58 ± 0.82 a	8.57 ± 0.41 b	10.83 ± 0.69 a	12.69 ± 0.28 a
Total SLs	119.7 ± 3.9 b	247.4 ± 17.8 a	137.4 ± 5.3 b	135.5 ± 18.9 b

The table only listed the HRMs and the summation of all individual HRMs detected in each class affected by Cu–pH interactions. Different letters behind the values in the same row represent a significant difference at *p* < 0.05. P3L, pH 3.0 + 0.5 μM Cu-treated leaves; P3CL: pH 3.0 + 300 μM Cu-treated leaves; P5L: pH 4.8 + 0.5 μM Cu-treated leaves; P5CL: pH 4.8 + 300 μM Cu-treated leaves. Total AUXs, CKs, ABAs, SAs, and SLs were the summation of all individual HRMs detected in each class.

**Table 2:** Impacts of Cu–pH interactions on mean (±SE, *n* = 3) concentrations (ng g^−1^ FW) of HRMs detected in *Citrus sinensis* roots.

Hormones and Related Metabolites		Treatments		
	P3R	P3CR	P5R	P5CR
CKs				
cis-Zeatin riboside (cZR)	0.481 ± 0.055 ab	0.373 ± 0.015 bc	0.585 ± 0.044 a	0.291 ± 0.013 c
2-Methylthio-N6-isopentenyladenosine (2MeSiPR)	4.760 ± 0.259 bc	7.322 ± 0.768 a	6.277 ± 0.544 ab	4.320 ± 0.309 c
6-Benzyladenosine (BAPR)	0.049 ± 0.008 b	0.024 ± 0.007 c	0.072 ± 0.003 a	0.008 ± 0.004 c
N6-Isopentenyl-adenine-9-glucoside (iP9G)	0.068 ± 0.034 b	0.187 ± 0.017 a	0.099 ± 0.008 b	0.212 ± 0.037 a
trans-Zeatin (tZ)	0.190 ± 0.001 b	0.295 ± 0.024 a	ND	0.201 ± 0.006 b
K9G	0.166 ± 0.033 a	0.077 ± 0.077 a	0.057 ± 0.057 a	ND
BAP9G	ND	0.221 ± 0.015 a	ND	0.022 ± 0.012 b
6-Benzyladenine (BAP)	ND	0.023 ± 0.023 b	0.069 ± 0.007 a	0.020 ± 0.010 b
2-Methylthio-N6-isopentenyladenine (2MeSiP)	0.005 ± 0.005 b	0.099 ± 0.010 a	ND	0.007 ± 0.007 b
IPR	1.015 ± 0.072 b	0.222 ± 0.016 c	3.097 ± 0.238 a	0.350 ± 0.017 c
cis-Zeatin-O-glucoside riboside (cZROG)	0.880 ± 0.009 b	1.013 ± 0.036 a	0.870 ± 0.009 b	0.926 ± 0.007 b
trans-Zeatin riboside (tZR)	1.292 ± 0.077 b	0.524 ± 0.026 d	1.867 ± 0.190 a	0.931 ± 0.055 c
ortho-Topolin (oT)	0.043 ± 0.005 ab	0.117 ± 0.046 a	0.036 ± 0.004 b	0.073 ± 0.014 ab
tZOG	3.044 ± 0.105 b	4.647 ± 0.419 a	4.515 ± 0.148 a	2.896 ± 0.031 b
IP	0.038 ± 0.003 b	0.697 ± 0.040 a	0.033 ± 0.006 b	0.086 ± 0.009 b
para-Topolin (pT)	0.034 ± 0.034 a	0.087 ± 0.044 a	ND	ND
cZ	ND	0.519 ± 0.004 a	ND	0.061 ± 0.006 b
BAP7G	ND	ND	0.001 ± 0.001	ND
Total CKs	12.58 ± 0.36 b	16.99 ± 0.82 a	18.11 ± 0.54 a	10.92 ± 0.31 b
AUXs				
2-oxindole-3-acetic acid (OxIAA)	12.192 ± 1.479 b	21.772 ± 2.530 a	7.893 ± 0.910 b	18.376 ± 1.023 a
IAA-Leu	0.176 ± 0.005	ND	ND	ND
Indole-3-acetyl-L-phenylalanne methyle ester (IAA-Phe-Me)	ND	0.062 ± 0.037	ND	ND
TRA	0.252 ± 0.027 b	9.115 ± 1.473 a	1.179 ± 0.185 b	0.737 ± 0.237 b
Indole-3-carboxylic acid (ICA)	1.665 ± 0.120 b	2.841 ± 0.293 a	2.931 ± 0.244 a	1.922 ± 0.147 b
IAA-Glu	2.856 ± 0.157 b	5.892 ± 0.728 a	2.432 ± 0.397 b	6.015 ± 0.726 a
Indole-3-acetyl glycine (IAA-Gly)	2.577 ± 0.581 ab	3.439 ± 0.141 a	2.123 ± 0.200 b	2.952 ± 0.091 ab
IAM	ND	ND	0.628 ± 0.099	ND
3-Indolepropionic acid (IPA)	ND	0.926 ± 0.194	ND	ND
TRP	4366 ± 120 c	7191 ± 450 a	5008 ± 312 bc	5636 ± 251 b
IAA	5.785 ± 0.164 c	11.558 ± 0.614 a	5.559 ± 0.301 c	8.672 ± 0.543 b
Indole-3-acetyl-L-aspartic acid (IAA-Asp)	20.13 ± 1.02 b	32.19 ± 2.42 a	16.17 ± 1.16 b	33.73 ± 3.12 a
IAA-Val	2.004 ± 0.069 a	1.390 ± 0.170 b	1.587 ± 0.051 b	1.354 ± 0.049 b
MEIAA	2.430 ± 0.146 b	9.057 ± 0.509 a	1.823 ± 0.046 b	2.346 ± 0.188 b
ICAld	8.813 ± 1.238 bc	22.580 ± 1.114 a	11.883 ± 1.414 b	7.546 ± 0.225 c
IAN	1.807 ± 0.150 b	1.631 ± 0.091 b	2.435 ± 0.230 a	1.985 ± 0.254 ab
Total AUXs	4433 ± 119 c	7319 ± 446 a	5070 ± 31 bc	5727 ± 256 b
JAs				
JA-ILE	22.50 ± 1.83 b	35.89 ± 1.33 a	34.27 ± 3.70 a	25.71 ± 1.59 b
Jasmonic acid (JA)	84.33 ± 2.53 b	120.75 ± 5.70 b	208.24 ± 42.52 a	157.77 ± 14.98 ab
3-oxo-2-(2-(Z)-Pentenyl) cyclopen-tane-1-hexanoic acid (OPC-6)	18.26 ± 0.52 a	ND	20.08 ± 1.93 a	ND
3-oxo-2-(2-(Z)-Pentenyl) cyclopen-tane-1-butyric acid (OPC-4)	16.44 ± 1.31 b	32.45 ± 2.70 a	27.20 ± 1.33 a	29.56 ± 2.95 a
OPDA	27.84 ± 2.30 b	48.15 ± 5.35 b	97.40 ± 20.46 a	40.13 ± 2.80 b
JA-Val	1.456 ± 0.123 b	1.250 ± 0.116 b	2.771 ± 0.386 a	1.381 ± 0.118 b
Total JAs	177.1 ± 5.3 b	245.8 ± 15.5 b	399.1 ± 68.2 a	261.0 ± 21.5 b
GAs				
Gibberellin A_7_ (GA_7_)	0.009 ± 0.009 a	0.005 ± 0.005 a	ND	ND
Gibberellin A_1_ (GA_1_)	28.006 ± 1.227 b	25.570 ± 2.717 b	39.848 ± 4.572 a	6.771 ± 1.411 c
GA_24_	0.351 ± 0.095 b	2.105 ± 0.601 a	0.690 ± 0.113 b	0.299 ± 0.108 b
Gibberellin A_9 (_GA_9_)	0.818 ± 0.136 b	1.849 ± 0.530 a	0.164 ± 0.164 b	0.667 ± 0.081 b
Total GAs	31.53 ± 1.47 b	32.87 ± 3.67 ab	42.76 ± 4.43 a	10.85 ± 1.33 c
ABAs				
ABA	1.769 ± 0.184 b	3.686 ± 0.137 a	3.267 ± 0.343 a	1.787 ± 0.223 b
Ethylene (ETH)				
1-Aminocyclopropanecarboxylic acid (ACC)	79.22 ± 1.69 ab	65.84 ± 4.93 bc	56.69 ± 1.56 c	95.64 ± 10.30 a
Salicylates (SAs)				
SAG	47.45 ± 1.55 b	255.51 ± 54.03 a	43.03 ± 0.64 b	75.47 ± 4.26 b
SA	45.05 ± 2.58 b	61.19 ± 7.92 a	60.35 ± 2.52 a	53.03 ± 0.75 ab
Total SAs	92.50 ± 3.40 b	316.70 ± 55.36 a	103.38 ± 1.98 b	128.50 ± 3.62 b
SLs				
ST	272.0 ± 11.7 b	262.7 ± 15.8 b	295.2 ± 18.6 b	378.0 ± 32.0 a

The table only listed the HRMs and the summation of all individual HRMs detected in each class affected by Cu–pH interactions. Different letters behind the values in the same row represent a significant difference at *p* < 0.05. P3R: pH 3.0 + 0.5 μM Cu-treated roots; P3CR: pH 3.0 + 300 μM Cu-treated roots; P5R: pH 4.8 + 0.5 μM Cu-treated roots; P5CR: pH 4.8 + 300 μM Cu-treated roots.

## Data Availability

Data are archived in L.-S. Chen’s lab and available upon request.

## Supplementary Materials

The following supporting information can be downloaded at: https://www.mdpi.com/article/10.3390/plants12112144/s1, Figure S1: Heatmap of 55 (56) hormones and related metabolites (HRMs) detected in *Citrus sinensis* leaves (roots); Figure S2: Principal component analysis (PCA) loading plots for 109 and 104 HRMs in pH 3.0-treated (A) and pH 4.8-treeated (B) leaves and roots, respectively at different Cu (0.5 and 300 μM) levels, for 107 and 104 HRMs in 300 (C) and 0.5 (D) μM Cu-treated leaves and roots, respectively at different pH (3.0, 4.0 and 4.8) levels, and for 55 and 56 HRMs in leaves (E) and roots (F), respectively; Table S1: List of 88 HRMs tested in *C. sinensis* leaves; Table S2: List of 88 HRMs tested in *C. sinensis* roots; Table S3: PCA for 109 HRMs in pH 3.0-treated leaves and roots; Table S4: PCA for 104 HRMs in pH 4.8-treated leaves and roots; Table S5: PCA for 107 HRMs in 300 μM Cu-treated leaves and roots; Table S6: PCA for 104 HRMs in 0.5 μM Cu-treated leaves and roots; Table S7: PCA for 55 HRMs in leaves; Table S8: PCA for 56 HRMs in roots; Table S9: Differentially abundant HRMs identified in P3L vs. P5L, P3CL vs. P3L, P5CL vs. P5L and/or P3CL vs. P5CL; Table S10: Differentially abundant HRMs identified in P3R vs. P5R, P3CR vs. P3R, P5CR vs. P5R and/or P3CR vs. P5CR.
