# Supplementary material for: Roles of Hormones in Elevated pH-Mediated Mitigation of Copper Toxicity in Citrus sinensis Revealed by Targeted Metabolome
Source: Plants (Basel). 2023 May 29;12(11):2144. doi: 10.3390/plants12112144 (PMC10255168; doi:10.3390/plants12112144)
Supplement: Supplementary file 1 [file plants-12-02144-s001.zip › 2023ZhangPlantsFigs. S1-S2 and Tabble S3-S100413.pdf]

Supplementary Figures S1-S2 and Tables S3-S10

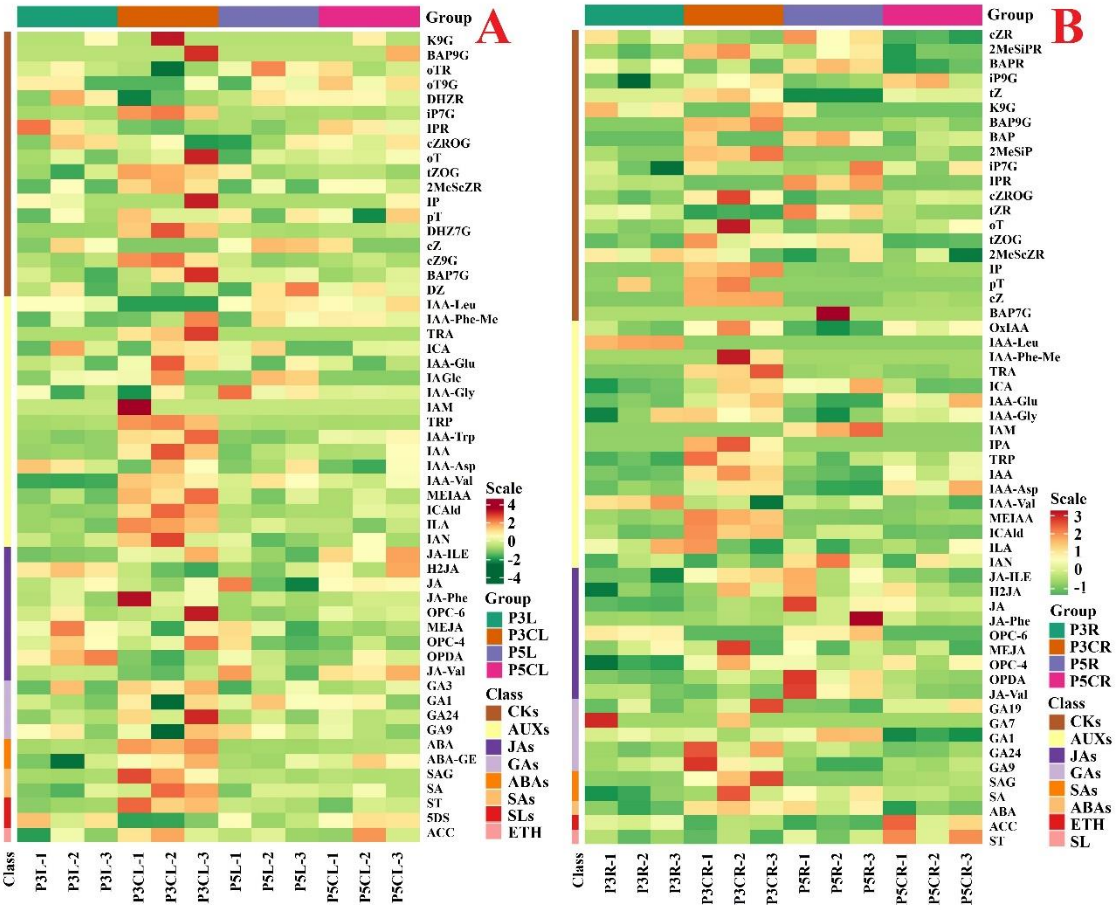

Figure S1. Heatmap of 55 (56) hormones and related metabolites (HRMs) detected in *Citrus sinensis* leaves (roots). P3L, pH 3.0 + 0.5 μM Cu-treated leaves; P3CL, pH 3.0 + 300 μM Cu-treated leaves; P5L, pH 4.8 + 0.5 μM Cu-treated leaves; P5CL, pH 4.8 + 300 μM Cu-treated leaves; P3R, pH 3.0 + 0.5 μM Cu-treated roots; P3CR, pH 3.0 + 300 μM Cu-treated roots; P5R, pH 4.8 + 0.5 μM Cu-treated roots; P5CR, pH 4.8 + 300 μM Cu-treated roots.

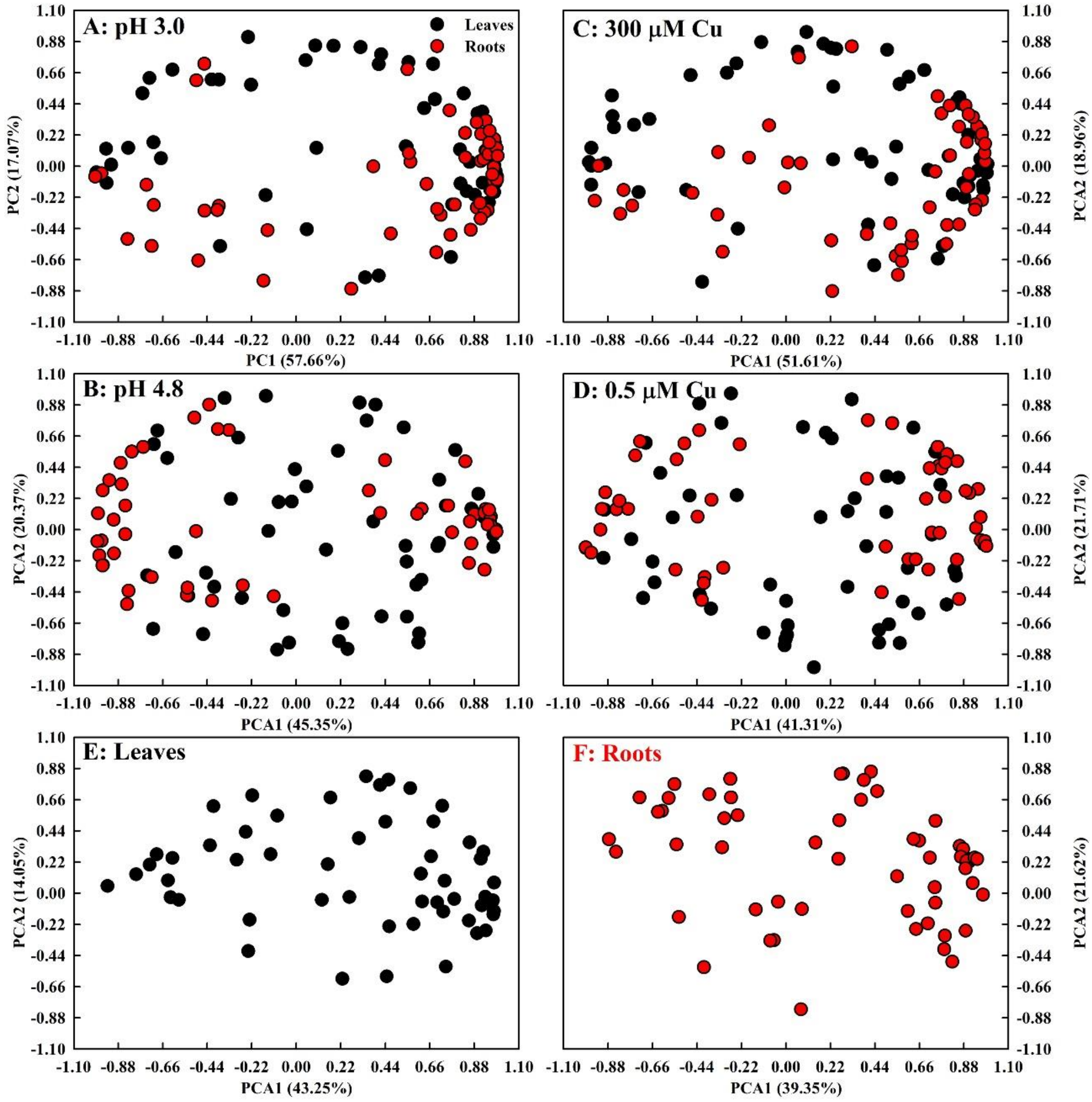

Figure S2. Principal component analysis (PCA) loading plots for 109 and 104 HRMs in pH 3.0-treated (A) and pH 4.8-treeated (B) leaves and roots, respectively at different Cu (0.5 and 300 μM) levels, for 107 and 104 HRMs in 300 (C) and 0.5 (D) μM Cu-treated leaves and roots, respectively at different pH (3.0, 4.0 and 4.8) levels, and for 55 and 56 HRMs in leaves (E) and roots (F), respectively.

Table S3. PCA for 109 HRMs in pH 3.0-treated leaves and roots

**Variables**	**PC1**	**PC2**	**PC3**	**PC4**	**PC5**
Leaf ABAs					
ABA	0.981	0.166	-0.100	-0.009	-0.030
ABA-GE	0.809	0.117	0.103	-0.309	-0.475
Leaf AUXs					
IAA-Leu	-0.989	-0.044	0.083	0.034	0.108
IAA-Phe-Me	0.408	0.719	0.501	0.255	0.012
TRA	0.896	0.372	0.207	-0.072	-0.106
ICA	0.100	0.128	0.629	0.759	0.044
IAA-Glu	0.842	-0.177	0.383	0.091	0.324
IAGlc	0.340	-0.787	0.051	0.510	-0.059
IAA-Gly	0.052	-0.447	0.735	-0.505	-0.056
IAM	0.380	-0.002	-0.921	-0.037	0.080
TRP	0.992	-0.075	-0.105	0.006	-0.011
IAA-Trp	0.916	0.359	0.065	-0.129	-0.100
IAA	0.958	-0.165	0.228	0.039	0.031
IAA-Asp	-0.152	-0.205	0.854	-0.102	0.442
IAA-Val	0.985	-0.036	-0.166	-0.027	0.000
MEIAA	0.919	0.384	-0.049	0.047	0.058
ICAld	0.983	-0.089	0.157	-0.030	0.000
ILA	0.979	0.003	-0.165	0.000	0.118
IAN	0.763	-0.643	-0.052	-0.021	0.021
Leaf CKs					
K9G	0.408	-0.774	0.438	0.148	-0.149
BAP9G	0.419	0.789	0.376	-0.129	-0.209
oTR	-0.761	0.514	-0.329	0.154	0.159
oT9G	-0.419	0.611	0.360	-0.111	0.556
DHZR	-0.706	0.168	0.331	0.588	-0.133
iP7G	0.986	-0.113	-0.103	0.024	0.056
IPR	-0.831	0.128	0.135	-0.310	0.422
cZROG	-0.377	-0.565	-0.130	0.717	-0.085
oT	0.554	0.734	0.389	0.037	0.034
tZOG	0.920	-0.117	-0.170	-0.187	-0.274
2MeScZR	0.857	0.029	-0.006	0.427	0.286
IP	0.184	0.849	0.439	-0.228	0.032
pT	0.541	0.139	-0.600	0.554	0.145
DHZ7G	0.978	-0.184	0.093	0.021	0.015
cZ	-0.669	0.055	0.019	0.735	-0.089
cZ9G	0.951	-0.256	-0.159	-0.056	-0.026
BAP7G	0.684	0.472	0.502	-0.215	0.104
DZ	-0.224	0.575	-0.061	0.394	0.679
Leaf ETH					
ACC	0.771	-0.272	-0.083	0.551	0.149
Leaf GAs					
GA_3	0.632	0.410	-0.053	0.561	0.339
GA_1	0.094	0.851	-0.436	-0.274	0.045
GA_{24}	0.676	0.721	-0.020	0.010	-0.151
GA_9	-0.239	0.911	-0.192	-0.098	0.259
Leaf JAs					
JA-ILE	0.828	0.513	0.131	-0.046	-0.181
H2JA	-0.938	-0.119	-0.239	0.170	0.140
JA	-0.383	0.610	-0.046	0.236	-0.651
JA-Phe	0.545	0.135	-0.799	0.062	0.206

OPC-6	0.317	0.840	0.432	0.078	0.038
MEJA	-0.613	0.680	-0.135	0.368	0.089
OPC-4	0.046	0.748	0.543	0.359	-0.122
OPDA	-0.941	0.122	0.022	0.182	-0.258
JA-Val	-0.727	0.622	0.217	0.028	-0.193
SAG	0.882	-0.203	-0.423	-0.005	0.048
Leaf SAs					
SA	0.811	-0.123	0.451	-0.050	-0.348
Leaf SLs					
ST	0.935	0.110	-0.334	0.002	-0.041
5DS	-0.916	0.010	0.165	-0.342	-0.132
Root ABAs					
Root ABA	0.975	-0.102	-0.056	-0.191	0.009
Root AUXs					
OxIAA	0.892	-0.293	0.224	-0.162	0.207
IAA-Leu	-0.992	-0.065	0.070	0.078	-0.019
IAA-Phe-Me	0.714	-0.344	0.606	0.066	0.017
TRA	0.936	0.321	0.087	-0.073	-0.087
ICA	0.912	0.035	0.355	0.132	-0.156
IAA-Glu	0.914	0.227	0.331	0.045	-0.048
IAA-Gly	0.564	0.030	-0.286	0.303	-0.712
IPA	0.945	-0.312	-0.059	0.034	0.067
TRP	0.926	0.049	-0.366	0.021	0.075
IAA	0.992	-0.096	0.065	-0.055	0.006
IAA-Asp	0.940	0.105	0.290	0.132	-0.060
IAA-Val	-0.835	-0.515	-0.125	0.133	-0.066
MEIAA	0.976	-0.009	-0.209	-0.063	-0.005
ICAld	0.950	0.084	-0.215	-0.206	0.045
ILA	-0.383	-0.281	-0.825	-0.094	-0.291
IAN	-0.391	-0.310	0.335	0.672	-0.433
cZR	-0.705	-0.275	-0.093	-0.645	-0.048
2MeSiPR	0.862	-0.450	-0.195	-0.113	0.059
BAPR	-0.715	-0.564	0.218	-0.318	-0.144
iP9G	0.835	0.061	0.117	-0.415	-0.336
tZ	0.930	-0.327	-0.146	0.044	0.071
Root CKs					
K9G	-0.494	0.605	0.420	-0.430	-0.168
BAP9G	0.979	0.190	-0.031	-0.051	-0.046
BAP	0.380	-0.002	-0.921	-0.037	0.080
2MeSiP	0.956	0.249	0.011	-0.155	-0.013
iP7G	0.557	0.091	-0.482	-0.359	0.566
IPR	-0.979	-0.068	0.117	-0.144	0.042
cZROG	0.911	-0.370	0.167	-0.066	0.015
tZR	-0.964	-0.052	0.140	0.096	0.199
oT	0.692	-0.609	0.370	0.014	0.117
tZOG	0.834	0.234	-0.494	0.017	0.068
2MeScZR	-0.485	-0.666	-0.534	-0.129	-0.138
IP	0.990	0.126	0.024	-0.044	-0.043
pT	0.466	-0.477	-0.259	0.541	0.442
cZ	0.995	0.071	-0.059	-0.032	-0.017
Root ETH					
ACC	-0.741	-0.132	0.623	-0.029	-0.210
Root GAs					
GA_{19}	0.549	0.683	0.444	0.187	-0.005

GA_7	-0.143	-0.453	0.334	-0.674	0.456
GA_1	-0.455	0.723	-0.467	0.220	0.064
GA_{24}	0.757	0.394	-0.482	-0.183	-0.078
GA_9	0.643	-0.127	-0.746	0.100	-0.053
Root JAs					
JA-ILE	0.953	0.166	0.130	-0.042	0.212
H2JA	0.782	-0.272	0.390	0.402	-0.017
JA	0.969	-0.060	0.206	-0.097	0.077
JA-Phe	0.696	-0.303	0.566	0.213	0.240
OPC-6	-0.994	-0.074	0.077	-0.025	0.017
MEJA	0.272	-0.867	0.213	0.342	-0.112
OPC-4	0.965	-0.177	0.096	0.165	-0.015
OPDA	0.908	-0.260	0.261	-0.100	0.170
JA-Val	-0.453	-0.314	0.485	0.116	0.668
Root SAs					
SAG	0.891	0.309	0.305	-0.069	-0.109
SA	0.763	-0.486	0.373	0.148	-0.148
Root SLs					
ST	-0.162	-0.810	0.372	-0.424	0.007
Eigen value	62.851	18.611	13.933	8.177	5.429
Variation percent (%)	57.661	17.074	12.782	7.502	4.981

Table S4. PCA for 104 HRMs in pH 4.8-treated leaves and roots

Variables	PC1	PC2	PC3	PC4	PC5
Leaf ABAs					
ABA	0.974	-0.123	-0.051	-0.115	0.140
ABA-GE	0.705	-0.095	-0.244	0.493	0.437
Leaf AUXs					
IAA-Leu	-0.064	-0.570	0.708	-0.372	0.178
IAA-Phe-Me	0.253	-0.843	-0.411	-0.046	-0.231
ICA	-0.139	-0.009	-0.288	-0.841	0.437
IAA-Glu	-0.324	0.216	0.654	0.611	-0.219
IAGlc	-0.708	-0.701	-0.062	-0.023	0.045
IAA-Gly	-0.687	0.699	0.166	0.061	0.093
TRP	-0.705	0.602	0.228	-0.042	0.294
IAA-Trp	0.974	-0.037	0.177	0.131	0.045
IAA	0.421	-0.614	0.495	0.088	0.440
IAA-Asp	-0.270	-0.483	0.701	0.283	0.351
IAA-Val	-0.461	-0.738	0.272	-0.392	0.124
MEIAA	-0.007	0.426	-0.104	0.829	0.348
ICAld	0.739	0.169	-0.219	0.201	0.581
ILA	-0.406	-0.404	0.573	-0.542	-0.223
IAN	0.347	0.769	-0.408	0.286	0.199
Leaf CKs					
K9G	0.415	0.117	-0.803	0.232	0.340
BAP9G	0.540	-0.114	0.644	-0.238	0.474
oTR	-0.534	-0.468	0.064	-0.422	-0.560
oT9G	0.607	-0.733	0.088	-0.054	-0.290
DHZR	-0.036	-0.799	-0.500	0.052	-0.330
iP7G	0.147	-0.142	0.631	-0.746	0.051
IPR	0.866	0.147	-0.159	0.267	-0.364
cZROG	0.547	-0.615	0.338	0.455	-0.036
oT	0.603	-0.796	0.010	-0.046	0.013
tZOG	-0.355	0.928	0.002	0.064	-0.090
2MeScZR	0.618	-0.354	-0.486	-0.387	-0.326
IP	0.540	-0.114	0.644	-0.238	0.474
pT	-0.088	0.194	0.953	0.204	-0.065
DHZ7G	0.707	0.352	0.420	-0.283	0.346
cZ	-0.737	-0.323	0.070	0.009	-0.589
cZ9G	0.312	0.896	-0.251	0.150	0.115
BAP7G	-0.597	-0.160	0.460	0.034	0.636
DZ	-0.095	-0.848	-0.232	0.453	0.117
Leaf ETH					
ACC	0.380	0.058	-0.772	-0.059	0.503
Leaf GAs					
GA_3	0.211	-0.788	0.288	0.369	0.339
GA_1	-0.446	-0.306	-0.735	-0.117	-0.392
GA_{24}	0.545	-0.227	-0.009	-0.566	-0.575
GA_9	-0.639	0.505	-0.137	-0.339	-0.452
Leaf JAs					
JA-ILE	0.899	0.252	0.273	-0.222	-0.066
H2JA	0.698	-0.116	0.697	0.111	0.036
JA	0.392	0.882	0.003	-0.262	-0.019
JA-Phe	0.593	-0.389	-0.346	-0.516	0.331
OPC-6	0.927	0.088	0.149	0.145	-0.298
MEJA	-0.287	0.649	0.012	-0.703	-0.052

OPC-4	-0.150	0.943	-0.175	-0.159	0.178
OPDA	0.206	0.556	-0.489	-0.304	0.563
JA-Val	0.531	0.721	0.160	-0.415	0.009
Leaf SAs					
SAG	0.049	0.305	-0.145	-0.825	-0.450
SA	0.228	-0.662	-0.656	0.282	0.031
Leaf SLs					
ST	-0.023	0.197	-0.223	-0.232	0.926
5DS	0.786	0.561	0.023	0.172	0.194
Root ABAs					
ABA	-0.864	0.320	0.071	0.166	0.344
Root AUXs					
OxIAA	0.961	0.091	0.157	0.144	-0.151
TRA	-0.542	-0.463	0.610	0.079	0.337
ICA	-0.902	-0.168	0.184	0.325	-0.138
IAA-Glu	0.935	0.145	0.316	-0.061	-0.011
IAA-Gly	0.880	0.108	0.255	0.381	0.062
IAM	-0.957	-0.251	0.065	0.095	0.089
TRP	0.618	0.146	0.628	0.444	-0.068
IAA	0.929	0.119	0.184	-0.076	-0.287
IAA-Asp	0.958	0.072	0.269	-0.057	-0.034
IAA-Val	-0.903	0.069	-0.140	0.399	-0.034
MEIAA	0.858	0.057	0.006	-0.146	0.488
ICAld	-0.837	-0.526	-0.066	0.043	0.129
ILA	0.438	0.490	0.718	0.061	0.218
IAN	-0.496	-0.012	-0.433	-0.542	0.522
cZR	-0.958	0.277	0.042	0.027	0.059
2MeSiPR	-0.830	-0.433	-0.014	0.096	0.339
BAPR	-0.963	-0.078	0.094	-0.173	0.165
iP9G	0.770	-0.019	-0.496	0.292	-0.274
tZ	0.987	0.001	-0.045	0.078	-0.135
Root CKs					
K9G	-0.431	0.881	0.129	-0.141	0.036
BAP9G	0.598	0.112	-0.727	0.278	-0.156
BAP	-0.846	-0.033	-0.023	-0.353	0.398
2MeSiP	0.415	0.117	-0.803	0.232	0.340
iP7G	-0.112	-0.470	0.639	0.590	0.101
IPR	-0.981	0.115	0.113	0.036	0.102
cZROG	0.931	-0.283	-0.146	0.111	0.143
tZR	-0.925	0.349	0.143	0.044	-0.019
oT	0.853	-0.237	0.273	-0.145	0.348
tZOG	-0.974	-0.183	0.049	-0.069	0.103
2MeScZR	-0.265	-0.395	-0.460	0.748	-0.051
IP	0.952	0.140	-0.095	-0.102	0.234
cZ	0.960	0.031	-0.246	0.126	-0.026
BAP7G	-0.417	-0.502	-0.268	-0.706	-0.059
Root ETH					
ACC	0.865	-0.098	0.201	0.001	-0.450
Root GAs					
GA_{19}	0.946	0.036	0.220	-0.187	0.144
GA_1	-0.958	-0.254	-0.046	-0.035	0.119
GA_{24}	-0.715	-0.336	0.176	-0.496	0.313
GA_9	0.836	0.481	0.250	-0.085	0.008
Root JAs					

JA-ILE	-0.757	0.583	0.108	0.230	-0.151
H2JA	-0.334	0.703	0.125	0.195	-0.584
JA	-0.504	0.790	0.247	0.115	-0.216
JA-Phe	-0.538	-0.411	0.224	0.701	0.001
OPC-6	-0.983	-0.083	0.093	0.092	0.094
MEJA	-0.388	0.710	-0.263	0.526	0.017
OPC-4	0.358	0.276	0.752	-0.111	-0.467
OPDA	-0.814	0.551	0.167	0.083	0.010
JA-Val	-0.869	0.471	0.138	-0.003	-0.066
Root SAs					
SAG	0.989	-0.015	0.066	-0.030	0.127
SA	-0.845	0.170	0.090	0.495	0.055
Root SLs					
ST	0.751	0.170	0.559	0.135	-0.277
Eigen value	47.162	21.181	14.900	11.510	9.247
Variation percent (%)	45.348	20.367	14.327	11.068	8.891

Table S5. PCA for 107 HRMs in 300 μM Cu-treated leaves and roots

**Variables**	**PC1**	**PC2**	**PC3**	**PC4**	**PC5**
Leaf ABAs					
Leaf ABA	0.964	0.252	-0.046	0.032	0.062
ABA-GE	0.234	0.561	0.391	-0.604	-0.336
Leaf AUXs					
IAA-Leu	-0.962	-0.132	0.031	0.147	-0.184
IAA-Phe-Me	-0.292	0.659	0.628	-0.177	0.234
TRA	0.846	0.457	0.259	0.004	0.091
ICA	0.372	0.084	0.767	-0.253	-0.450
IAA-Glu	0.825	-0.200	0.425	0.292	0.114
IAGlc	0.751	-0.654	0.072	0.020	-0.050
IAA-Gly	-0.237	-0.443	0.852	-0.096	-0.112
IAM	0.423	0.030	-0.900	0.086	0.061
TRP	0.997	0.014	-0.050	0.035	0.044
IAA-Trp	0.685	0.677	0.224	0.142	-0.041
IAA	0.877	-0.120	0.338	0.169	-0.271
IAA-Asp	0.521	-0.092	0.659	0.357	-0.397
IAA-Val	0.911	-0.065	-0.215	0.312	-0.149
MEIAA	0.858	0.486	-0.015	-0.149	0.070
ICAld	0.949	-0.032	0.255	-0.130	-0.126
ILA	0.961	0.050	-0.117	0.240	0.044
IAN	0.775	-0.565	-0.037	-0.278	-0.030
Leaf CKs					
K9G	0.436	-0.700	0.445	-0.331	-0.110
BAP9G	0.059	0.808	0.394	0.263	-0.346
oTR	-0.750	0.291	-0.299	0.336	0.387
oT9G	-0.857	0.353	0.301	0.206	0.091
DHZR	-0.893	0.020	0.399	-0.165	0.126
iP7G	0.993	-0.048	-0.053	0.091	0.015
IPR	-0.962	0.002	0.059	-0.087	0.254
cZROG	-0.414	-0.818	-0.123	0.328	-0.193
oT	0.249	0.827	0.502	0.041	0.028
tZOG	0.980	0.030	-0.120	-0.022	0.156
2MeScZR	0.883	-0.221	0.068	-0.173	0.371
IP	0.186	0.864	0.420	0.141	-0.152
pT	0.233	0.047	-0.352	0.895	-0.138
DHZ7G	0.974	-0.135	0.168	0.054	-0.042
cZ	-0.493	-0.169	0.073	0.371	0.765
cZ9G	0.977	-0.164	-0.118	-0.052	0.038
BAP7G	0.562	0.578	0.581	0.017	-0.104
DZ	-0.850	0.274	-0.154	-0.422	0.013
Leaf ETH					
ACC	0.407	-0.414	-0.103	-0.753	-0.293
Leaf GAs					
GA_3	0.863	0.442	-0.047	0.149	-0.186
GA_1	-0.245	0.725	-0.417	-0.240	0.428
GA_{24}	0.500	0.821	0.050	0.128	0.239
GA_9	-0.122	0.876	-0.239	0.026	0.401
Leaf JAs					
JA-ILE	-0.471	0.642	0.333	0.472	-0.180
H2JA	-0.728	-0.183	-0.272	0.524	-0.296
JA	-0.860	0.499	0.001	-0.107	-0.032
JA-Phe	0.545	0.137	-0.826	0.040	0.002

OPC-6	0.222	0.834	0.476	0.045	0.159
MEJA	0.101	0.946	-0.257	0.130	0.106
OPC-4	0.607	0.630	0.402	-0.266	0.039
OPDA	-0.675	0.332	-0.079	-0.551	-0.352
JA-Val	-0.962	0.128	0.094	0.125	-0.186
Leaf SAs					
SAG	0.903	-0.144	-0.394	0.074	0.064
SA	0.705	-0.027	0.654	-0.266	0.071
Leaf SLs					
ST	0.901	0.220	-0.318	-0.099	-0.168
5DS	-0.974	0.028	0.110	-0.115	-0.161
Root ABAs					
ABA	0.985	0.031	-0.029	-0.118	-0.117
Root AUXs					
OxIAA	0.621	-0.546	0.476	0.298	-0.006
IAA-Phe-Me	0.711	-0.292	0.638	-0.028	-0.028
TRA	0.888	0.428	0.150	0.052	0.056
ICA	0.805	0.071	0.518	0.109	0.259
IAA-Glu	-0.083	0.287	0.696	0.554	-0.346
IAA-Gly	0.769	0.371	-0.427	0.274	-0.118
IPA	0.970	-0.239	-0.014	0.032	0.018
TRP	0.812	0.075	-0.475	0.329	0.022
IAA	0.892	-0.152	0.252	0.318	0.126
IAA-Asp	-0.182	0.059	0.643	0.632	-0.387
IAA-Val	0.229	-0.883	-0.364	-0.155	0.106
MEIAA	0.982	0.092	-0.158	0.033	0.012
ICAld	0.966	0.185	-0.165	0.016	0.066
ILA	-0.007	-0.152	-0.896	0.356	-0.219
IAN	-0.463	-0.190	0.164	-0.614	-0.588
cZR	0.905	-0.056	-0.307	-0.169	0.234
2MeSiPR	0.939	-0.271	-0.154	-0.045	-0.141
BAPR	0.797	-0.416	0.212	0.115	-0.365
iP9G	-0.335	0.098	0.305	-0.653	0.599
tZ	0.935	-0.308	-0.099	0.093	0.113
Root CKs					
K9G	0.326	0.845	0.408	-0.026	0.109
BAP9G	0.945	0.282	0.022	-0.079	0.142
BAP	0.074	0.019	-0.938	-0.037	-0.335
2MeSiP	0.925	0.345	0.046	-0.131	0.077
iP7G	0.010	0.025	-0.645	0.726	-0.237
IPR	-0.926	0.001	0.216	-0.013	-0.310
cZROG	0.856	-0.413	0.280	-0.064	-0.118
tZR	-0.945	-0.245	0.135	0.056	0.160
oT	0.543	-0.635	0.445	0.082	-0.311
tZOG	0.856	0.278	-0.425	0.068	0.065
2MeScZR	0.516	-0.404	-0.478	-0.478	0.336
IP	0.970	0.224	0.084	0.011	0.046
pT	0.793	-0.549	-0.258	0.048	-0.021
cZ	0.985	0.158	-0.006	0.003	0.073
Root ETH					
ACC	-0.802	-0.170	0.327	0.405	0.238
Root GAs					
GA_{19}	0.064	0.766	0.598	0.129	-0.186
GA_7	0.573	-0.672	0.463	-0.017	-0.077

GA_1	0.903	0.365	-0.194	-0.096	0.062
GA_{24}	0.751	0.493	-0.428	0.098	0.028
GA_9	0.738	-0.039	-0.655	0.158	-0.002
Root JAs					
JA-ILE	0.894	0.171	0.222	-0.005	0.349
H2JA	0.224	-0.526	0.616	0.118	0.529
JA	-0.759	-0.280	0.270	0.258	0.454
JA-Phe	-0.338	-0.343	0.359	0.433	0.672
MEJA	0.554	-0.769	0.213	-0.235	0.047
OPC-4	0.400	-0.481	0.399	0.666	0.081
OPDA	0.623	-0.493	0.507	0.073	0.326
JA-Val	-0.313	-0.605	0.486	0.172	0.521
Root SAs					
SAG	0.810	0.427	0.400	0.002	0.040
SA	0.570	-0.594	0.561	-0.083	-0.009
Root SLs					
ST	-0.819	-0.337	0.159	0.434	-0.046
Eigen value	55.218	20.284	16.649	8.517	6.332
Variation percent (%)	51.605	18.957	15.560	7.960	5.918

Table S6. PCA for 104 HRMs in 0.5 μM Cu-treated leaves and roots

Variables	PC1	PC2	PC3	PC4	PC5
Leaf ABAs					
ABA	-0.706	-0.483	0.474	0.027	-0.206
ABA-GE	0.498	0.377	-0.585	-0.228	-0.464
Leaf AUXs					
IAA-Leu	0.794	-0.530	-0.045	-0.041	0.292
IAA-Phe-Me	0.563	-0.802	0.180	-0.064	-0.051
ICA	-0.078	-0.388	0.892	-0.216	-0.015
IAA-Glu	0.306	0.129	-0.109	0.933	0.082
IAGlc	0.460	-0.709	-0.046	-0.232	-0.480
IAA-Gly	0.630	0.719	-0.182	0.019	0.229
TRP	0.226	0.644	0.388	-0.059	-0.616
IAA-Trp	-0.243	0.242	-0.843	0.394	-0.131
IAA	0.509	-0.669	-0.444	0.162	-0.264
IAA-Asp	-0.426	-0.460	-0.541	0.535	0.168
IAA-Val	0.978	-0.104	-0.014	-0.183	0.004
MEIAA	0.496	0.124	0.147	0.843	-0.078
ICAld	0.739	0.549	-0.245	0.295	0.072
ILA	0.841	-0.327	0.200	-0.101	0.368
IAN	-0.428	0.890	0.007	0.155	-0.033
Leaf CKs					
K9G	-0.476	0.240	-0.099	-0.439	-0.716
oTR	0.655	-0.594	0.305	-0.290	0.203
oT9G	-0.006	-0.817	-0.179	0.159	0.524
DHZR	0.009	-0.677	0.495	0.008	-0.545
iP7G	0.603	-0.272	0.636	-0.124	0.379
IPR	-0.765	-0.068	-0.262	0.278	0.515
cZROG	-0.370	-0.559	0.002	0.259	-0.696
oT	0.138	-0.973	0.040	0.118	0.137
tZOG	0.325	0.920	-0.010	-0.153	-0.158
2MeScZR	0.006	-0.744	0.629	-0.134	0.182
IP	-0.659	-0.227	-0.162	0.298	0.632
pT	0.340	0.221	0.320	0.778	-0.357
DHZ7G	0.750	0.550	0.360	-0.064	0.047
cZ	0.578	-0.509	0.429	0.035	-0.471
cZ9G	-0.272	0.961	0.002	-0.032	-0.039
BAP7G	0.722	-0.036	-0.244	0.314	0.566
DZ	0.461	-0.799	-0.183	0.340	-0.029
Leaf ETH					
ACC	0.305	-0.405	0.843	0.028	-0.176
Leaf GAs					
GA_3	-0.002	-0.775	0.292	0.538	-0.154
GA_1	0.834	-0.286	-0.046	-0.262	0.391
GA_{24}	0.001	-0.505	0.732	-0.457	0.006
GA_9	0.171	0.088	0.547	0.248	0.776
Leaf JAS					
JA-ILE	0.198	0.683	0.655	-0.191	0.172
H2JA	-0.902	-0.200	-0.062	0.350	-0.139
JA	-0.319	0.753	0.572	-0.040	0.054
JA-Phe	-0.111	-0.729	0.525	0.075	0.420
OPC-6	-0.650	-0.374	0.368	0.515	0.192
MEJA	-0.559	0.085	0.796	-0.005	0.216
OPC-4	-0.621	0.400	0.633	0.226	0.045

OPDA	-0.896	0.140	0.319	-0.162	-0.222
JA-Val	0.085	0.724	0.617	-0.108	0.278
Leaf SAs					
SAG	0.556	0.366	0.337	-0.654	0.126
SA	0.398	-0.118	-0.433	-0.534	-0.596
Leaf SLs					
ST	0.764	0.315	0.505	-0.213	-0.128
5DS	-0.693	0.612	-0.356	-0.004	0.138
Root ABAs					
ABA	0.847	0.483	-0.105	0.189	0.054
Root AUXs					
OxIAA	-0.780	0.146	-0.344	0.327	0.381
IAA-Leu	-0.989	-0.129	-0.031	0.022	-0.064
TRA	0.964	-0.076	-0.213	0.062	-0.128
ICA	0.940	0.012	-0.065	0.172	-0.286
IAA-Glu	-0.540	0.496	0.576	0.351	0.086
IAA-Gly	-0.368	0.210	0.069	-0.058	-0.901
IAM	0.984	-0.082	-0.123	0.036	-0.091
TRP	0.693	0.217	-0.001	0.671	-0.148
IAA	-0.428	0.702	0.137	-0.270	0.482
IAA-Asp	-0.838	0.143	0.482	0.180	-0.113
IAA-Val	-0.917	0.000	-0.151	0.060	-0.364
MEIAA	-0.910	0.146	-0.291	-0.240	0.094
ICAld	0.704	-0.282	-0.589	-0.155	0.231
ILA	-0.745	0.523	-0.149	-0.064	-0.382
IAN	0.721	-0.021	0.481	-0.493	-0.075
cZR	0.525	0.751	-0.322	0.004	0.238
2MeSiPR	0.846	-0.214	-0.468	-0.111	-0.087
BAPR	0.787	0.232	-0.332	-0.459	0.072
iP9G	0.401	0.359	-0.701	-0.444	-0.150
tZ	-0.990	-0.125	-0.059	0.026	-0.022
Root CKs					
K9G	-0.721	0.623	-0.026	0.043	0.300
BAP	0.963	0.087	0.150	-0.185	0.094
2MeSiP	-0.437	0.091	-0.585	0.006	0.677
iP7G	0.606	-0.210	-0.540	0.528	0.134
IPR	0.950	0.286	-0.001	0.125	0.015
cZROG	-0.310	-0.270	-0.830	-0.377	-0.020
tZR	0.757	0.451	0.253	0.370	0.150
oT	-0.402	-0.334	-0.664	-0.337	0.416
tZOG	0.991	-0.115	0.066	0.005	0.006
2MeScZR	-0.546	-0.283	-0.655	0.151	-0.414
IP	-0.502	0.608	0.243	-0.517	0.227
pT	-0.415	-0.498	0.600	0.469	0.017
BAP7G	0.474	-0.443	0.173	-0.706	0.225
Root ETH					
ACC	-0.963	-0.163	-0.145	-0.136	-0.087
Root GAs					
GA_{19}	-0.406	-0.377	0.763	0.313	0.112
GA_7	-0.437	0.091	-0.585	0.006	0.677
GA_1	0.856	-0.491	-0.093	-0.079	-0.106
GA_{24}	0.759	-0.024	-0.220	-0.576	0.206
GA_9	-0.893	0.262	0.273	0.022	-0.244
Root JAs					

JA-ILE	0.770	0.431	0.167	0.393	0.198
H2JA	0.711	0.433	0.457	0.276	-0.147
JA	0.751	0.584	0.205	0.217	0.080
JA-Phe	0.642	-0.210	-0.470	0.453	-0.342
OPC-6	0.494	-0.120	-0.695	0.439	-0.258
MEJA	-0.229	0.602	0.256	0.324	-0.644
OPC-4	0.907	0.258	0.303	0.086	-0.109
OPDA	0.797	0.533	0.160	0.221	0.082
JA-Val	0.788	0.474	0.248	0.242	0.182
Root SAs					
SAG	-0.823	0.202	-0.300	-0.435	0.040
SA	0.889	0.273	-0.011	0.093	-0.357
Root SLs					
ST	0.405	0.772	-0.350	0.341	0.024
Eigen value	42.961	22.578	17.372	10.720	10.369
Variation percent (%)	41.309	21.710	16.703	10.307	9.971

Table S7. PCA for 55 HRMs in leaves

Variables	PC1	PC2	PC3	PC4	PC5	PC6	PC7	PC8	PC9
ABAs									
ABA	0.977	0.074	-0.061	-0.100	0.126	0.021	-0.018	-0.062	-0.030
ABA-GE	0.615	0.137	-0.001	0.225	-0.605	0.169	0.056	-0.139	-0.333
AUXs									
IAA-Leu	-0.934	0.050	0.181	0.058	-0.165	0.064	-0.036	0.170	0.014
IAA-Phe-Me	0.168	0.674	0.598	0.185	-0.178	0.001	0.287	-0.002	0.013
TRA	0.924	0.292	-0.037	0.077	0.110	-0.137	-0.054	-0.074	-0.069
ICA	0.155	0.204	0.116	0.379	0.454	-0.246	0.578	0.284	0.054
IAA-Glu	0.726	-0.130	0.112	0.243	0.126	-0.128	-0.379	0.258	0.282
IAGlc	0.228	-0.605	0.615	0.039	0.291	-0.270	0.088	0.050	-0.140
IAA-Gly	-0.231	-0.188	-0.098	0.425	-0.531	-0.558	-0.206	0.203	0.169
IAM	0.459	-0.235	-0.128	-0.802	0.042	0.232	-0.045	-0.092	-0.065
TRP	0.976	-0.128	-0.054	-0.076	0.121	-0.036	-0.031	-0.025	0.007
IAA-Trp	0.856	0.358	-0.075	0.080	-0.174	0.294	-0.039	-0.014	-0.056
IAA	0.916	-0.088	0.189	0.262	0.009	0.133	-0.096	0.089	-0.008
IAA-Asp	0.126	-0.048	0.213	0.536	0.537	-0.050	-0.567	-0.027	0.056
IAA-Val	0.695	-0.065	0.392	-0.203	-0.476	-0.088	0.019	0.231	-0.008
MEIAA	0.911	0.243	-0.012	-0.113	-0.003	-0.044	-0.041	0.002	0.018
ICAld	0.972	-0.051	-0.032	0.193	-0.066	0.053	0.039	-0.023	0.059
ILA	0.945	-0.056	0.104	-0.203	0.040	-0.052	-0.094	0.095	0.079
IAN	0.739	-0.520	-0.319	0.140	-0.014	0.073	0.105	-0.076	0.174
CKs									
K9G	0.446	-0.589	-0.026	0.600	0.025	0.018	0.278	-0.078	0.064
BAP9G	0.413	0.763	-0.056	0.241	-0.060	0.106	-0.166	0.159	-0.283
oTR	-0.691	0.274	0.391	-0.440	-0.066	-0.188	0.037	0.141	0.033
oT9G	-0.410	0.613	0.427	0.120	-0.077	0.353	-0.106	-0.105	0.291
DHZR	-0.725	0.201	0.379	0.210	0.171	0.008	0.383	0.150	-0.111
iP7G	0.975	-0.141	-0.016	-0.076	0.103	0.006	-0.029	0.018	0.060
IPR	-0.613	0.247	-0.244	0.143	0.098	0.400	-0.160	-0.352	0.381
cZROG	-0.238	-0.409	0.330	0.148	0.323	0.644	0.078	0.216	-0.183
oT	0.563	0.740	0.287	0.183	0.050	0.092	0.016	-0.066	-0.043
tZOG	0.852	-0.195	-0.312	-0.126	-0.194	-0.237	-0.043	0.052	-0.085
2MeScZR	0.733	0.087	0.237	-0.050	0.100	0.264	0.430	0.027	0.310
IP	0.345	0.824	-0.112	0.227	0.201	-0.021	-0.253	-0.094	-0.091
pT	0.262	-0.027	0.010	-0.344	0.086	0.185	-0.422	0.744	-0.067
DHZ7G	0.973	-0.151	-0.024	0.115	0.021	0.001	-0.004	0.060	0.077
cZ	-0.581	-0.047	0.504	-0.199	0.159	-0.348	0.105	0.313	-0.042
cZ9G	0.936	-0.265	-0.203	-0.053	0.036	0.015	0.003	-0.048	0.055
BAP7G	0.677	0.506	0.139	0.288	-0.078	-0.313	-0.232	-0.003	0.061
DZ	-0.295	0.236	0.806	-0.125	-0.065	0.169	0.026	-0.149	0.079
ETH									
ACC	0.579	-0.218	0.106	0.052	-0.054	0.191	0.651	0.006	0.166
GAs									
GA_3	0.665	0.260	0.383	-0.109	0.443	0.252	0.023	0.132	0.036
GA_1	-0.094	0.546	0.231	-0.607	-0.342	-0.237	0.114	-0.282	-0.005
GA_{24}	0.722	0.616	-0.015	-0.188	0.058	-0.009	0.067	-0.017	-0.130
GA_9	-0.218	0.690	-0.132	-0.512	0.108	-0.374	-0.059	-0.032	0.184
JAs									
JA-ILE	0.440	0.503	-0.205	0.114	-0.445	0.417	0.112	0.311	0.019
H2JA	-0.621	-0.030	-0.154	-0.004	0.317	0.630	-0.240	0.162	-0.058
JA	-0.127	0.274	-0.818	0.017	-0.157	0.049	0.311	0.321	0.051
JA-Phe	0.622	-0.059	-0.051	-0.695	0.124	0.273	0.060	-0.091	0.032

OPC-6	0.455	0.801	-0.064	0.198	0.258	0.056	-0.024	-0.036	0.026
MEJA	-0.250	0.433	-0.423	-0.295	0.601	-0.246	0.187	0.137	0.030
OPC-4	0.309	0.387	-0.533	0.173	0.517	-0.331	0.164	0.034	0.011
OPDA	-0.634	0.090	-0.440	0.098	0.451	0.008	0.279	-0.167	-0.259
JA-Val	-0.427	0.337	-0.445	0.069	-0.462	0.143	0.230	0.428	0.144
SAs									
SAG	0.893	-0.283	-0.070	-0.315	0.046	0.070	-0.001	-0.019	0.044
SA	0.780	-0.042	0.197	0.447	-0.176	-0.106	0.148	-0.142	-0.178
SLs									
ST	0.934	-0.026	-0.094	-0.261	0.013	0.000	0.068	-0.032	-0.101
5DS	-0.792	0.133	-0.408	0.268	-0.187	0.223	-0.078	-0.143	-0.046
Eigen value	23.786	7.726	5.091	4.534	3.763	3.062	2.609	1.894	1.024
Variation percent (%)	43.247	14.046	9.257	8.243	6.841	5.568	4.744	3.443	1.862

Table S8. PCA for 56 HRMs in roots

Variables	PC1	PC2	PC3	PC4	PC5	PC6	PC7	PC8
ABAs								
ABA	0.421	0.857	0.108	0.045	-0.016	0.161	-0.012	0.087
AUXs								
OxIAA	0.889	-0.265	-0.148	0.261	0.036	0.031	0.135	0.168
IAA-Leu	-0.405	-0.523	0.673	0.179	-0.171	0.123	-0.002	-0.078
IAA-Phe-Me	0.712	0.250	0.052	0.511	-0.346	-0.179	0.035	-0.027
TRA	0.860	0.332	0.161	-0.214	-0.243	0.057	0.069	-0.077
ICA	0.282	0.844	-0.189	0.092	-0.131	-0.054	0.174	-0.297
IAA-Glu	0.786	-0.301	-0.487	-0.039	-0.101	0.083	-0.001	0.044
IAA-Gly	0.701	-0.214	0.040	-0.083	0.248	0.188	-0.230	-0.451
IAM	-0.551	0.769	-0.162	-0.078	0.080	-0.169	0.150	-0.096
IPA	0.876	0.311	0.276	0.196	0.071	-0.054	-0.073	0.072
TRP	0.865	0.256	-0.085	-0.172	0.334	0.171	0.015	0.041
IAA	0.974	-0.011	-0.153	0.078	0.010	0.014	-0.017	0.102
IAA-Asp	0.783	-0.396	-0.444	0.008	-0.015	0.027	-0.028	0.031
IAA-Val	-0.529	-0.169	0.670	0.435	0.153	0.074	-0.079	-0.107
MEIAA	0.910	0.226	0.326	-0.073	0.034	0.044	-0.051	0.056
ICAld	0.739	0.510	0.363	-0.189	0.014	-0.051	0.114	0.068
ILA	-0.059	-0.332	0.410	-0.062	0.604	0.426	-0.236	0.124
IAN	-0.541	0.344	-0.283	-0.030	-0.111	-0.437	-0.469	-0.007
cZR	-0.613	0.580	0.345	0.128	-0.100	0.276	0.037	0.165
2MeSiPR	0.451	0.719	0.350	0.124	0.221	-0.256	0.079	0.078
BAPR	-0.631	0.573	0.344	0.188	-0.117	-0.097	0.006	0.123
iP9G	0.602	-0.126	-0.468	-0.055	0.015	-0.099	0.146	-0.098
tZ	0.823	-0.484	0.246	0.129	0.071	0.031	-0.016	0.025
CKs								
K9G	-0.149	-0.115	0.409	-0.103	-0.687	0.536	0.049	0.063
BAP9G	0.923	0.242	0.198	-0.139	-0.130	0.042	-0.003	-0.055
BAP	-0.272	0.676	-0.133	-0.385	0.406	-0.109	-0.251	0.226
2MeSiP	0.893	0.227	0.258	-0.164	-0.199	0.049	0.048	-0.006
iP7G	0.146	0.357	-0.239	-0.219	0.532	0.056	0.617	0.157
IPR	-0.723	0.675	-0.067	0.026	-0.034	0.091	0.025	0.005
cZROG	0.924	0.071	0.036	0.279	0.032	-0.207	0.039	0.072
tZR	-0.874	0.380	-0.109	0.132	-0.067	0.207	-0.029	0.028
oT	0.736	0.041	-0.061	0.539	0.011	-0.287	0.013	0.185
tZOG	0.271	0.841	0.218	-0.369	0.145	-0.022	-0.036	0.008
2MeScZR	-0.039	-0.062	0.755	0.256	0.342	-0.063	0.252	-0.287
IP	0.932	0.250	0.199	-0.085	-0.141	0.032	-0.010	-0.015
pT	0.549	0.119	0.428	0.324	0.280	-0.089	-0.240	0.039
cZ	0.946	0.241	0.191	-0.076	-0.054	0.022	-0.020	-0.005
BAP7G	-0.316	0.324	-0.022	-0.284	-0.145	-0.709	-0.216	0.217
ETH								
ACC	0.075	-0.821	-0.415	0.160	0.013	-0.007	0.158	-0.027
GAs								
GA_{19}	0.643	-0.254	-0.322	-0.311	-0.462	0.032	0.020	-0.139
GA_7	0.079	-0.113	0.397	0.477	-0.209	-0.023	0.434	0.590
GA_1	-0.379	0.697	0.512	-0.187	-0.118	-0.128	0.066	-0.125
GA_{24}	0.659	0.370	0.324	-0.541	0.070	0.107	-0.030	0.073
GA_9	0.739	-0.069	0.415	-0.114	0.363	0.230	-0.263	0.079
JAs								
JA-ILE	0.386	0.798	-0.190	0.038	-0.170	0.329	0.024	0.039
H2JA	0.264	0.514	-0.568	0.416	-0.071	0.184	-0.200	-0.161

JA	-0.239	0.549	-0.667	0.109	0.034	0.381	-0.120	0.092
JA-Phe	-0.305	0.528	-0.164	0.064	0.292	-0.046	0.587	-0.394
OPC-6	-0.840	0.291	0.382	0.103	-0.094	0.054	0.121	-0.072
MEJA	0.260	0.242	0.235	0.829	0.103	0.064	-0.276	-0.177
OPC-4	0.631	0.382	-0.604	0.137	0.147	-0.004	-0.100	0.034
OPDA	-0.275	0.806	-0.348	0.155	-0.042	0.323	-0.106	0.090
JA-Val	-0.580	0.671	-0.267	0.172	-0.090	0.267	-0.099	0.112
SAs								
SAG	0.888	0.174	0.064	-0.115	-0.377	0.026	0.078	-0.096
SA	0.372	0.659	-0.310	0.511	0.010	-0.139	-0.033	-0.167
SLs								
ST	-0.077	-0.336	-0.799	0.221	0.287	0.147	0.154	0.165
Eigen value	22.036	12.105	7.241	3.840	2.949	2.323	1.927	1.427
Variation percent (%)	39.349	21.616	12.931	6.857	5.267	4.148	3.440	2.548

Table S9. Differentially abundant HRMs identified in P3L vs. P5L, P3CL vs. P3L, P5CL vs. P5L and/or P3CL vs. P5CL

Hormones	Fold change			
	P3L vs. P5L	P3CL vs. P3L	P5CL vs. P5L	P3CL vs. P5CL
CKs				
K9G	Inf		Inf	
BAP9G		Inf	Inf	
DHZR		0.529		0.539
iP7G		4.192		3.662
IPR	2.458	0.298	2.247	0.326
tZOG		1.336		1.285
2MeScZR		4.221		
IP	Inf		Inf	
DHZ7G		11.741		4.564
cZ		0.000		0.000
cZ9G		3.130		2.881
BAP7G		1.949		
Total CKs		1.399		1.330
AUXs				
IAA-Leu		0		0
TRA		Inf		Inf
IAA-Glu		3.525		3.586
IAGlc			0	Inf
IAM		Inf		Inf
TRP		8.375		9.434
IAA-Trp		3.937	2.742	1.565
IAA		2.188		1.681
IAA-Val	0.726	1.492		1.150
MEIAA		2.237		1.783
ICAld		1.941		1.557
ILA		1.893		1.726
IAN		1.753		
Total AUXs		7.962		8.963
JAs				
JA-ILE		3.412	2.969	
H2JA	1.372	0.659		0.712
OPDA	1.670	0.433		
JA-Val				0.344
GAs				
GA_{24}		1.334		
ABAs				
ABA		5.305		4.647
ABA-GE		2.813		
Total ABAs		2.983		
SAs				
SAG		8.100		5.570
SA		1.478		
Total SAs		3.036		2.482

SLs				
ST		2.228		1.945
5DS		0.681		0.675
Total SLs		2.066		1.826

A HRM was considered downregulated or upregulated when it had both a fold change of less or more, respectively, than 1 and a $P < 0.05$. Also, a HRM was considered downregulated or upregulated when it was detected only in the control sample (fold change = 0) or treated sample for a comparative group. Inf, hormone was detected only in treated sample for a comparative group.

Table S10. Differentially abundant HRMs identified in P3R vs. P5R, P3CR vs. P3R, P5CR vs. P5R and/or P3CR vs. P5CR

Hormones	Fold change			
	P3R vs. P5R	P3CR vs. P3R	P5CR vs. P5R	P3CR vs. P5CR
CKs				
cZR			0.498	
2MeSiPR		1.538	0.688	1.695
BAPR	0.681	0.497	0.113	
iP9G		2.757	2.149	
tZ	Inf	1.555	Inf	1.469
K9G			0	Inf
BAP9G		Inf	Inf	10.128
BAP	0	Inf	0.287	
2MeSiP	Inf	19.038	Inf	13.430
IPR	0.328	0.218	0.113	
cZROG		1.150		1.094
tZR	0.692	0.406	0.499	0.563
tZOG	0.674	1.527	0.642	1.604
IP		18.302		8.139
pT	Inf			Inf
cZ		Inf	Inf	8.541
BAP7G	0		0	
Total CKs	0.695	1.350	0.603	1.556
AUXs				
OxIAA		1.786	2.328	
IAA-Leu	Inf	0		
IAA-Phe-Me		Inf		Inf
TRA		36.215		12.369
ICA	0.568	1.706	0.656	1.479
IAA-Glu		2.063	2.473	
IAM	0		0	
IPA		Inf		Inf
TRP		1.647		1.276
IAA		1.998	1.560	1.333
IAA-Asp		1.599	2.086	
IAA-Val	1.263	0.694		
MEIAA		3.727		3.860
ICAld		2.562	0.635	2.992
IAN	0.742			
Total AUXs		1.651		1.278
JAs				
JA-ILE	0.657	1.595		1.396
JA	0.405			
OPC-6		0	0	
OPC-4	0.604	1.974		
OPDA	0.286		0.412	
JA-Val	0.526		0.498	
Total JAs	0.444		0.654	
GAs				
GA_7	Inf			Inf
GA_1	0.703		0.170	3.776
GA_{24}		5.993		7.038

GA_9		2.259		2.770
Total GAs	0.737		0.254	3.030
SAs				
SAG		5.385		3.386
SA	0.747	1.358		
Total SAs		3.424		2.465
ABAs				
ABA	0.541	2.084	0.547	2.062
ETH				
ACC	1.397		1.687	0.688
SLs				
ST			1.281	0.695
